# SBMLsqueezer: A CellDesigner plug-in to generate kinetic rate equations for biochemical networks

**DOI:** 10.1186/1752-0509-2-39

**Published:** 2008-04-30

**Authors:** Andreas Dräger, Nadine Hassis, Jochen Supper, Adrian Schröder, Andreas Zell

**Affiliations:** 1 Center for Bioinformatics Tübingen (ZBIT), University of Tübingen, Sand 1, 72076 Tübingen, Germany

## Abstract

**Background:**

The development of complex biochemical models has been facilitated through the standardization of machine-readable representations like SBML (Systems Biology Markup Language). This effort is accompanied by the ongoing development of the human-readable diagrammatic representation SBGN (Systems Biology Graphical Notation). The graphical SBML editor CellDesigner allows direct translation of SBGN into SBML, and vice versa. For the assignment of kinetic rate laws, however, this process is not straightforward, as it often requires manual assembly and specific knowledge of kinetic equations.

**Results:**

SBMLsqueezer facilitates exactly this modeling step via automated equation generation, overcoming the highly error-prone and cumbersome process of manually assigning kinetic equations. For each reaction the kinetic equation is derived from the stoichiometry, the participating species (e.g., proteins, mRNA or simple molecules) as well as the regulatory relations (activation, inhibition or other modulations) of the SBGN diagram. Such information allows distinctions between, for example, translation, phosphorylation or state transitions. The types of kinetics considered are numerous, for instance generalized mass-action, Hill, convenience and several Michaelis-Menten-based kinetics, each including activation and inhibition. These kinetics allow SBMLsqueezer to cover metabolic, gene regulatory, signal transduction and mixed networks. Whenever multiple kinetics are applicable to one reaction, parameter settings allow for user-defined specifications. After invoking SBMLsqueezer, the kinetic formulas are generated and assigned to the model, which can then be simulated in CellDesigner or with external ODE solvers. Furthermore, the equations can be exported to SBML, LaTeX or plain text format.

**Conclusion:**

SBMLsqueezer considers the annotation of all participating reactants, products and regulators when generating rate laws for reactions. Thus, for each reaction, only applicable kinetic formulas are considered. This modeling scheme creates kinetics in accordance with the diagrammatic representation. In contrast most previously published tools have relied on the stoichiometry and generic modulators of a reaction, thus ignoring and potentially conflicting with the information expressed through the process diagram. Additional material and the source code can be found at the project homepage (URL found in the Availability and requirements section).

## Background

For modeling and simulating biochemical networks the machine-readable Systems Biology Markup Language (SBML) [1,2] is recognized as the standard format. SBML is a well-defined XML-based document structure with the objective of exchanging biological models. Numerous SBML-based programs for creating, manipulating or simulating kinetic equations are available, for instance SBMLeditor [3], the SBML ODE Solver Library (SOSlib) [4], COPASI [5], or the SBToolbox for Matlab™ [6-8]. To facilitate the modeling of biochemical networks, a growing number of programs provide diagrammatic interfaces and are capable of translating these into SBML [9]. Among these is also CellDesigner [10,11], which is, according to a survey [12], the most popular stand-alone application for modeling and simulating biochemical networks.

To simulate the dynamic behavior of these biochemical networks, kinetic equations have to be associated with each reaction. If the reaction mechanism is known, the kinetic formula can be derived from generalized mass-action kinetics [13]. Otherwise, a generic kinetic equation can be utilized, such as the recently published convenience rate law [14]. The derived formulas can be assigned to each reaction in several ways, for instance by manual input of C-like strings or through the selection of kinetic equations from predefined lists. In any case, the inserted formulas should be in agreement with the SBML and SBGN representation of the model. Hence, either the user is required to assure this consistency or an automated procedure is needed which assigns kinetic equations consistently with the SBGN representation.

To bridge the gap between the SBGN and systems of kinetic equations, SBMLsqueezer was developed. This CellDesigner plug-in allows one to specify the quantitative dynamics of biological networks, i.e., metabolic, signal transduction and gene regulatory networks based on the SBGN. Thereby, it distinguishes between different reaction types such as transcription, translation or state transition, between different species, e.g., simple molecules, proteins, genes or mRNA as well as between different regulatory modes like activation or inhibition. SBMLsqueezer takes all this information into account and provides a contextual selection of possible formulas for each particular reaction within the model. For each reaction in these networks a specific or a generic kinetic equation can be applied. The resulting equations are written directly into the SBML file as Math ML [15] strings such that the user can simulate the model directly within CellDesigner. Earlier approaches towards automatic equation generation like Cellerator [16,17] require the user to generate a new model while choosing rate equations for each reaction step-by-step from a predefined list of kinetics. In other approaches the same type of generic equation is assigned to each reaction [18]. The framework COPASI [5] supports the import of SBML files, and allows the user to select a kinetic formula for each reaction from a drop-down list. This drop-down list is generated in accordance with the stoichiometry and the number of modulators. JDesigner [19,20] provides a graphical representation and also allows the selection of rate laws for each reaction from a limited list.

Complementing these efforts, SBMLsqueezer extracts reaction-specific information directly from the CellDesigner-SBML file without the need for user interaction while respecting the annotations expressed through the CellDesigner process diagram, thus ensuring a coherent SBGN and SBML representation. This process depends on the annotations associated with each reactant, reaction and modulation. Such annotations are well-defined within CellDesigner and were only recently incorporated into the SBML specifications in the form of Systems Biology Ontology (SBO) [21].

### Implementation

Our work is based on the current *β*-version 4.0 of CellDesigner, as it provides an interface for plug-in development. SBMLsqueezer is written completely in Java and, in contrast to earlier approaches [16-18], only depends on freely available software. Furthermore, the Application Programming Interface (API) is available from the homepage [22], which allows inclusion of SBMLsqueezer as an equation generation module into other applications.

## Results and Discussion

SBMLsqueezer covers various kinetic models. To decide which kinetic equation to apply to a particular reaction, each reaction is analyzed for its properties, such as reactants, products and all participating modulators.

Non-enzyme state transition reactions are modeled through generalized mass-action kinetics. Whenever this equation can be applied, SBMLsqueezer also offers the zeroth order forward or reverse mass-action kinetics, depending on the reversibility property of the reaction. SBMLsqueezer covers all special cases of this type of equation defined in the SBO besides a few irreversible rates for discrete simulation. It also allows for non-integer stoichiometries.

For enzyme-catalyzed uni-uni reactions, the user can specify whether Michaelis-Menten or convenience kinetics should be assigned. Enzymatic reactions with more reaction partners, for instance bi-bi reactions, may either be modeled using convenience or detailed ternary-complex kinetics with different reaction mechanisms, for instance random, ordered or ping-pong. For bi-uni mechanisms the kinetic equations were manually derived using the King-Altman method [23-25] (see Additional file 1 – SBMLsqueezer: Kinetic Laws). If the number of reactants or products exceeds two, then convenience kinetics is applied, which is not restricted by the number of products or reactants. All other enzyme kinetics rate laws from the SBO are implemented in SBMLsqueezer as well and appear as an alternative choice whenever the structure and the context of the reaction is adequate for the particular formula.

For gene regulatory networks, i.e., transcriptional and translational processes, the Hill equation is applied [13,26]. SBMLsqueezer sets the boundary condition of genes automatically so that their amount cannot decrease because of transcriptional processes. If no transcriptional or translational activator participates, a basal reaction rate is assumed using a zeroth order mass-action rate law.

To incorporate control mechanisms, activation and inhibition terms were derived and integrated into the respective kinetic equations (see Additional file 1 – SBMLsqueezer: Kinetic Laws).

Since there is no specification of enzymes in the current version of CellDesigner, SBMLsqueezer allows the user to select molecule types that can act as biocatalysts. Generic and truncated proteins, RNA and complex molecules are accepted by default, but can also be deactivated. Additionally, simple and unknown molecules, 'asRNA' and receptors may in some cases be useful as enzymes. For metabolic networks, enzymatic reactions without an explicit catalyst are permitted to be treated as enzyme reactions.

Reactions with more than two reactants are unlikely to take place, therefore warnings will be given for those reactions. However, they can, depending on the context, be modeled using convenience or mass-action kinetics. Warnings are also indicated for unrealistic reactions, e.g., if transcriptional activation is assigned to a protein phosphorylation or if transcription and translation are used improperly.

Furthermore, SBMLsqueezer allows setting all reactions in the SBML file to reversible to generate all equations in a reversible manner. As Cornish-Bowden points out, this feature is often required: &ldquoModels for multi-enzyme systems must always take account of effects of products, because there is no way to ensure that product concentrations are zero in the conditions of interest [[25], pp. 312–313].&rdquo

In cases where specific equations were already assigned to some reactions SBMLsqueezer allows the user to specify if these equations should be overwritten or left unchanged.

**Figure 1:**
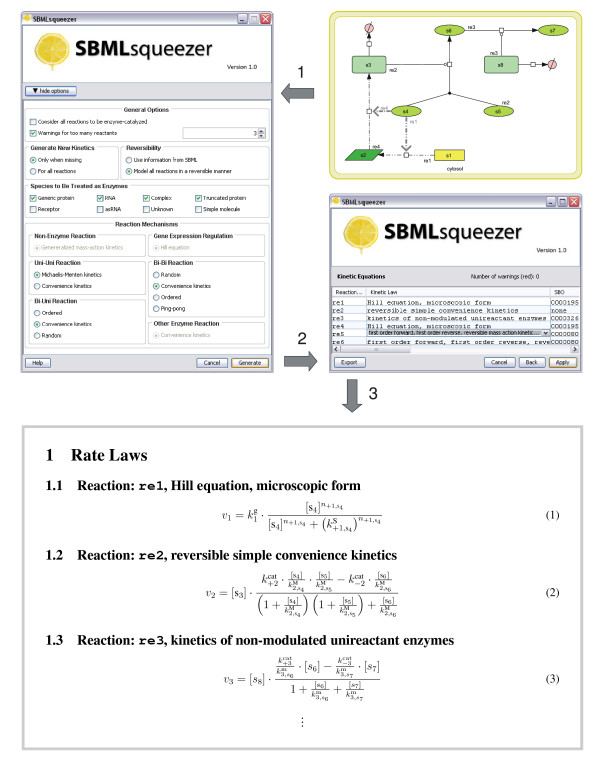
**The SBMLsqueezer work flow**. This graphic depicts the progression through SBMLsqueezer. The first step is to construct or open a diagrammatic model in CellDesigner. Then SBMLsqueezer can be started through the plug-in menu (step 1). The first window shows the standard kinetics and enables the user to specify the type of kinetics to be applied whenever there are multiple choices. By clicking on &ldquoGenerate&rdquo (step 2), the kinetics are compiled and presented. If necessary, warnings are indicated along with the respective formula. The kinetic equations summarized in the table can be altered by double-clicking on the name of the equation, which is taken from the SBO. SBMLsqueezer then offers a list of all applicable kinetic equations for the given reaction. Subsequently, all kinetics can be assigned to the SBML model and exported to plain text or LaTeX.

After specifying the parameter settings, SBMLsqueezer can be invoked to generate all equations. These are displayed in a comprehensive list, which the user can alter by double-clicking on the name of any formula (see Figure 1). A specific pull-down menu containing all applicable kinetic formulas for this particular reaction allows modification of the kinetic equations of the reaction network according to the biological knowledge of the user (see Additional file 2 – SBMLsqueezer: Tutorial). The names presented in this menu are given by the SBO terms for each kinetic equation whenever the SBO contains a definition of the particular formula. In these cases the SBO identifier number also appears in the table.

**Figure 2:**
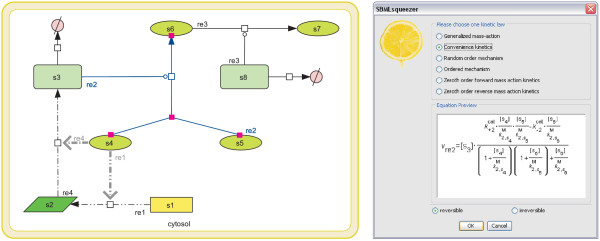
**Modeling reactions one by one using the context menu**. SBMLsqueezer provides a context menu which enables the user to create kinetic equations for the model one by one. Therefore, SBMLsqueezer analyzes the structure of the reaction and offers a selection of possible rate laws for the desired reaction. The names shown in this dialog window are generic terms. The tool tips associated with each option present the detailed SBO term for the particular choice. An equation preview [34] facilitates selection of an appropriate rate equation. Furthermore, SBMLsqueezer provides a radio button to set the reaction as reversible or irreversible possibly changing the selection of available rate laws.

Alternatively, kinetic formulas can be assigned separately to each reaction. Therefore, SBMLsqueezer provides an entry in CellDesigner's reaction context menu (see Figure 2). When called upon, SBMLsqueezer analyzes the particular reaction and provides a list of all kinetic equations that can be assigned to the given reaction and allows setting the reaction to be reversible or irreversible. When altering the reversibility property the selection of available equations may change. For the sake of simplicity, the context menu always shows the most generic names of each kinetic equation. Tool tips present the exact name of the rate law to be assigned according to the SBO or literature annotation.

After defining all rate equations, the kinetic parameters can be estimated with respect to measurement data, which often involves model optimization [12,27-29], or can be collected from the literature and databases [30-32]. Initially, the parameters and product concentrations, in absence of user-defined values, are set to one. In contrast to the default values of zero, this allows the model to be simulated directly in CellDesigner and permits the application of a model optimization procedure.

An export function allows storing complete information about the SBML model in a comprehensive LaTeX file. After compiling to a human-readable format like PDF, an overview of all model properties is provided, including initial values of the species, parameter values, event assignments, rate laws for each species and so forth. This overview may be used to assist model development and scientific writing. This function can also be applied to models that were created with other applications and already existing kinetic equations.

## Conclusion

SBMLsqueezer allows one to automatically assign kinetic equations in accordance with the SBGN employed by CellDesigner, such that the dynamic behavior of the model can be simulated over time. The majority of the kinetic formulas described in the SBO are covered, and complemented by additional formulas, for instance convenience kinetics.

Even though the annotation information of CellDesigner is helpful when deriving kinetic rates from SBGN models, many of these annotations are CellDesigner-specific and do not follow a standard format. Such a standard specification of annotations for reactants and reactions has recently been published in the form of the SBO. This specification allows one to distinguish between detailed reaction mechanisms, i.e., whether a uni-uni enzyme reaction has a competitive or a non-competitive inhibition mechanism. However, some reaction mechanisms (e.g., various bi-bi enzyme reactions) still cannot be distinguished. Hence, further refinements of the SBO would be beneficial in this context. This enables the modeler to specify exact reaction mechanisms as soon as they become integrated into the SBML-based programs. Accordingly, programs for automatic kinetic information will benefit from this wealth of information and thus will add rigor and consistency to the still complex modeling process.

## Availability and Requirements

The current version of SBMLsqueezer is contained within this article (see Additional file 3 – SBMLsqueezer.jar), and is also available through the project homepage [22].

Project name: SBMLsqueezer

Project homepage: [22]

Operating Systems: SBMLsqueezer was successfully tested under Linux (kernel 2.6.9), Mac OS X (10.5.1) and Windows XP

Programming Language: Java™

Other Requirements: CellDesigner 4.0 [33] and Java Runtime Environment 1.5

License: Creative Commons Attribution-Noncommercial-Share Alike 2.0 Germany License 

## Authors' contributions

JS and AD developed the conceptual idea. AD and NH implemented the software tool. AS provided detailed biochemical knowledge, a model of the T-cell signaling cascade as a modeling example and wrote the supplementary tutorial. AD and JS wrote this manuscript. AD wrote the supplementary material &ldquoKinetic Laws.&rdquo NH derived the formula for the bi-uni mechanisms random order and ordered. This work was supervised by AZ. All authors read and approved the final manuscript.

## Supplementary Material

Additional file 1 **SBMLsqueezer: Kinetic Laws**. This supplement provides a complete list of all currently supported kinetic laws together with conventions according to the graphical notation of CellDesigner and points out all special cases that are considered for an automatic equation generation including all available SBO terms for mathematical expressions. The derivation of the formula for the rapid equilibrium random order ternary-complex mechanism with one product as well as the ordered ternary-complex bi-uni mechanism can also be found in this document. Click here for file

Additional file 2 **SBMLsqueezer: Tutorial**. This tutorial provides installation instructions and guides the user through a real-world example of a T-cell signaling network. It highlights the modeling capabilities and the application of SBMLsqueezer to create a quantitative biochemical model. Finally it is shown how to apply CellDesigner's simulation module to the network to produce a dynamic simulation of the temporal changes of the species' concentrations. Click here for file

Additional file 3 **SBMLsqueezer.jar**. This is the current version (1.0) of the    SBMLsqueezer plug-in for CellDesigner. Click here for file
