# Supplementary material for: SBMLsqueezer: A CellDesigner plug-in to generate kinetic rate equations for biochemical networks
Source: BMC Syst Biol. 2008 Apr 30;2:39. doi: 10.1186/1752-0509-2-39 (PMC2412839; doi:10.1186/1752-0509-2-39)
Supplement: Additional file 1 — SBMLsqueezer: Kinetic Laws. This supplement provides a complete list of all currently supported kinetic laws together with conventions according to the graphical notation of CellDesigner and points out all special cases that are considered for an automatic equation generation including all available SBO terms for mathematical expressions. The derivation of the formula for the rapid equilibrium random order ternary-complex mechanism with one product as well as the ordered ternary-complex bi-uni mechanism can also be found in this document. [file 1752-0509-2-39-S1.pdf]

EBERHARD-KARLS-UNIVERSITÄT TÜBINGEN
Wilhelm-Schickard-Institut für Informatik
Lehrstuhl Rechnerarchitektur

Supplementary Material

SBMLsqueezer: Kinetic Laws

Andreas Dräger* Nadine Hassis Jochen Supper
Adrian Schröder Andreas Zell

April 28, 2008

*Corresponding author, e-mail: andreas.draeger@uni-tuebingen.de

Contents

1. Extracting Specific Rate Laws from SBML Process Diagrams **3**

2. Supported Kinetic Equations **5**
- 2.1. Generalized Mass-Action Kinetics 6
- 2.2. Uni-Uni Michaelis-Menten Kinetics 7
- 2.3. Bi-Uni Enzyme Reactions 9
  - 2.3.1. Random Order Mechanism 9
  - 2.3.2. Ordered Mechanism 10
- 2.4. Bi-Bi Enzyme Reactions 10
  - 2.4.1. Random Order Mechanism 11
  - 2.4.2. Ordered Mechanism 12
  - 2.4.3. Ping-Pong Mechanism 14
- 2.5. Irreversible Non-Modulated Non-Interacting Reactant Enzymes 14
- 2.6. Convenience Kinetics and Thermodynamics 15
  - 2.6.1. Thermodynamically Dependent Form 16
  - 2.6.2. Thermodynamically Independent Form 16
- 2.7. Hill Equation 17

3. Derivation of Predefined Kinetic Equations for Bi-Uni Reactions **18**
- 3.1. Ordered Bi-Uni Mechanism 18
- 3.2. Random Order Bi-Uni Mechanism 22

A. Systems Biology Ontology of Mathematical Expressions **25**

References **36**

1. Extracting Specific Rate Laws from SBML Process Diagrams

SBMLsqueezer was designed to interpret process diagrams created by CellDesigner and to apply rate laws for each reaction depending on the context. This document describes how SBMLsqueezer extracts the desired information from process diagrams and gives an overview of all currently supported rate laws. Each rate law is explained with an example process diagram, the general formula and the result yielded by SBMLsqueezer. At the end of each section we discuss the Systems Biology Ontology (SBO) terms which correspond to the introduced rate law. The appendix of this document contains a table of all currently available SBO terms for mathematical expressions in cellular systems (Section A, page 25).

Models of biological systems consist of reacting species. The rate of change of each species' concentration is determined by the reactions it is involved in. This rate depends on the velocities of the reactions. Each reaction velocity can basically be influenced by two types of modifiers: inhibitors and activators. Inhibitors lower the reaction velocity whereas activators speed up or enable the reaction. Any interaction of modulators with the reaction must be reflected in the rate law. An activated molecule can be an activator but this is not necessarily true. Here activation means activation of a reaction, in contrast to the activation of a molecule, which is a reaction in which the non-activated form of the molecule turns into the active form.

The graphical notation of process diagrams used by CellDesigner extends standard SBML [1] with additional information that can be interpreted to automatically assign appropriate rate laws to each reaction. CellDesigner allows including reaction-specific information to only a certain level of detail, thus several reaction mechanisms cannot be distinguished. For instance, it is not possible to include exact formulas for inhibition and activation as can be done for selected mechanisms in relevant text books like those of Cornish-Bowden or Segel [2, 3]. Often the process diagrams do not show at which state the modifier affects the reaction, i. e., whether the inhibitor reacts with the first substrate or the second one, with the ES_1, the ES_2 or with the EP_1P_2 complex, and so on.

To overcome this difficulty, Liebermeister and Klipp defined a generic inhibition and activation term [4]. This function is a prefactor, that can be multiplied with a kinetic equation to introduce modification. SBMLsqueezer applies this prefactor also to rather more detailed rate laws like the random order, ping-pong or the ordered ternary-complex mechanism.

CellDesigner 4.0α supports specialized arrows for two types of activating modification: one for transcriptional and another one for translational activation. A specific arrow for activation of enzyme reactions has been available since version 4.0β: the trigger symbol and—depending on the context—the symbol for physical stimulation. We reached the following accommodation: Besides the trigger and physical stimulation symbol, SBMLsqueezer also interprets an unknown catalysis arrow as an activation of the respective reaction (Table 1). This also allows modeling an activation with the α-version of CellDesigner 4.0. For a complete list of all symbols used in CellDesigner reference can be made to Kitano *et al.* [5] and the CellDesigner homepage [6]. The term "Modulation" was also introduced

Table 1: Redefinition of unknown catalysis to cover activation in process diagrams

Graphical Notation	CellDesigner Convention	SBMLsqueezer Interpretation
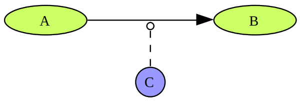	Unknown Catalysis	Activation
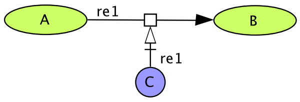	Trigger	Activation
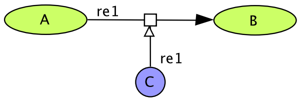	Physical Stimulation	Activation
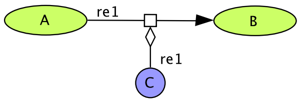	Modulation	Activation and Inhibition

by CellDesigner 4.0β. It specifies one of the two kinds of interplay between reaction and modulator. Hence its meaning is decidedly not clear. By considering such a modulator as both inhibitor and activator SBMLsqueezer assumes that in a later parameter optimization process an appropriate optimizer determines which role prevails.

In some cases, certain rate laws are special cases of other ones that only differ in their parameter settings. In these cases SBMLsqueezer always assigns the most general equation to the reaction, driven by the assumption that in a later parameter optimization process an optimizer will find the correct solution. Alternatively, all rate laws created by SBMLsqueezer may also be modified manually using the designated CellDesigner dialog boxes.

2. Supported Kinetic Equations

This section gives a complete list of all currently supported kinetic formulas and shows examples in the graphical notation of CellDesigner [7, 5, 6]. For an up-to-date list, refer to the project web page `http://www.ra.cs.uni-tuebingen.de/software/SBMLsqueezer` [8].

According to the process diagram, it often remains unclear at which state of the reaction the inhibitor or activator binds to enzyme, substrate or some intermediate complex. As stated in Section 1, SBMLsqueezer applies a generic formula for inhibition and activation for many rate laws such as the generalized mass-action kinetics or the rather detailed ternary-complex mechanisms. Equation (1), which was defined by Liebermeister *et al.* [4] in the context of convenience kinetics (Section 2.6, page 15), gives the general formula for this prefactor of the desired rate equation:

$$f_j(\mathbf{S},\mathbf{p}) = \prod_m h^{\mathrm{A}}([\mathrm{S}_m], k^{\mathrm{A}}_{jm})^{w^+_{jm}} h^{\mathrm{I}}([\mathrm{S}_m], k^{\mathrm{I}}_{jm})^{w^-_{jm}}. \tag{1}$$

where $\mathbf{S}$ and $\mathbf{p}$ are vectors of the concentrations of all reacting species in the system or parameter values, respectively. The matrices $\mathbf{N}^{\pm}$ contain the absolute values of all positive or negative elements of the stoichiometric matrix $\mathbf{N}$ or zero otherwise [4]. The matrices $\mathbf{W}^{\pm}$ are derived from the modulation matrix $\mathbf{W}$ in a similar way [4]. The modulation functions read:

$$h_{\mathrm{A}}([\mathrm{S}_m], k^{\mathrm{A}}_{jm}) = \frac{[\mathrm{S}_m]}{k^{\mathrm{A}}_{jm} + [\mathrm{S}_m]} \tag{2}$$

$$h_{\mathrm{I}}([\mathrm{S}_m], k^{\mathrm{I}}_{jm}) = \frac{k^{\mathrm{I}}}{k^{\mathrm{I}}_{jm} + [\mathrm{S}_m]}. \tag{3}$$

As an alternative to this simplified approach, one has to include all possible parameters assuming for a single inhibitor that it potentially acts at each state during the reaction. If more detailed knowledge about the mechanism is known, the rate law generated by SBMLsqueezer may serve as an initial equation that can be modified manually.

For reactions with two or more catalysts, one rate law will be generated for each catalyst. The rate law for this particular reaction is given as the sum of the rates of all participating catalysts. If the reaction is one of those, whose modification is modeled according to Equation (1), the whole rate law will be multiplied by the modification term f.

$$v_j(\mathbf{S},\mathbf{p}) = f_j(\mathbf{S},\mathbf{p}) \cdot \sum_{c=1}^{|\text{catalysts}|} v_{jc}(\mathbf{S},\mathbf{p}) \tag{4}$$

We also grant that the enzyme may be omitted from the process diagram for the sake of simplicity and clear arrangement of the reacting species. SBMLsqueezer therefore offers a checkbox asking whether all reactions should be considered as being enzyme-catalyzed. In this case, all factors $[\mathrm{E}]_0 \cdot k^{\mathrm{cat}}_{\pm}$ are replaced by the parameters $V^{\mathrm{m}}_{\pm}$ that hide the enzyme concentration and allow estimation of the whole factor by appropriate optimizers.

The context menu of SBMLsqueezer for single reactions considers RNA and asRNA, simple and unknown molecules, complexes, truncated as well as generic proteins, and receptors all as enzymes to allow the user to apply any possible kinetic formula to a certain reaction whereas the plug-in window provides user settings to restrict this list to a more detailed selection of possible enzymes.

In SBML every species has an identifier (ID) and may also have a name. The ID is an obligatory tag whereas the name may be empty [1, 9]. The name is intended to be a biologically meaningful identifier, which can in some cases be very long. Since the ID is supposed to be a short systematic identifier, SBMLsqueezer uses the ID for its LaTeX export.

2.1. Generalized Mass-Action Kinetics

In SBMLsqueezer generalized mass-action kinetics utilizes Equation (1) to include modification effects. This approach has already been successfully applied [10]. Figure 1 depicts an example of a

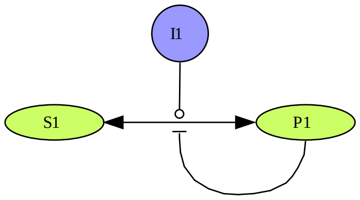

Figure 1: Example of a reaction to be modeled using the generalized mass-action rate law

reaction which is catalyzed by ion I_1 and thus cannot be modeled using enzyme kinetic approaches. This reaction may have an arbitrary mechanism in which the product P_1 acts as an inhibitor. Equation (5) shows the general formula for the reversible, and Equation (6) for the irreversible, case. The reaction velocity v_j of reaction j depends on a vector of all reacting species $\mathbf{S}$ and a parameter vector $\mathbf{p}$. Equation (7) gives the kinetic equation generated for the process in Figure 1:

$$v_j(\mathbf{S},\mathbf{p}) = F_j(\mathbf{S},\mathbf{p}) \cdot \left(k_{+j} \prod_i [\mathrm{S}_i]^{n_{ij}^-} - k_{-j} \prod_i [\mathrm{S}_i]^{n_{ij}^+} \right) \tag{5}$$

$$v_j(\mathbf{S},\mathbf{p}) = F_j(\mathbf{S},\mathbf{p}) \cdot k_{+j} \prod_i [\mathrm{S}_i]^{n_{ij}^-} \tag{6}$$

$$v_1 = \frac{k^{\mathrm{I}}_{1,\mathrm{P1}}}{k^{\mathrm{I}}_{1,\mathrm{P1}} + [\mathrm{P}_1]} \cdot \left(k_{+1}[\mathrm{S}_1][\mathrm{I}_1] - k_{-1}[\mathrm{P}_1][\mathrm{I}_1] \right). \tag{7}$$

$F_j(\mathbf{S},\mathbf{p})$ is allowed to be any positive function [11]. Thus all kinetic equations presented in the remainder of this section are special forms of this general formula as they can be derived from Equation (5) by setting $F_j(\mathbf{S},\mathbf{p})$ appropriately. Because of the availability of more specific equations, SBMLsqueezer restricts F_j in the following way:

$$F_j(\mathbf{S},\mathbf{p}) = f_j(\mathbf{S},\mathbf{p}). \tag{8}$$

Generalized mass-action kinetics allows modeling of reactions with any number of reactant and product molecules. However, since reactions with more than two reactants are unlikely to take place [2], warnings will be displayed.

SBMLsqueezer applies Equations (5) and (6) to all reactions which are not catalyzed by an enzyme or catalyzed by non-enzymes. Gene regulation (transcription) processes that are neither activated nor inhibited by other factors proceed at a constant rate (basal gene expression) and hence follow a zeroth order mass-action rate law. Another example of where SBMLsqueezer applies mass-action kinetics is in degradation processes.

The Systems Biology Ontology (SBO) defines several special cases of generalized mass-action kinetics (Table 2). None of the formulas defined therein includes the presence of any modulators. Monoexpotential decay (SBO:0000333) as a special case of first order irreversible mass-action kinetics (SBO:0000049) is indirectly supported by SBMLsqueezer because the rate constant k_{+j} can

be set to a value less than one. SBMLsqueezer was designed to create rate laws for continuous simulators and does not support any derivatives of irreversible mass-action kinetics, discrete scheme (SBO:0000166). Note that the discrete formulas SBO:0000140[1], SBO:0000141[2], SBO:0000143[3] and SBO:0000146[4] are formally identical to their corresponding continuous forms. All other special cases of the mass-action kinetics in SBO can be created by SBMLsqueezer. Whenever a mass-action rate law is applicable, SBMLsqueezer also offers selection of a zeroth order rate law (either for the forward or the reverse reaction).

2.2. Uni-Uni Michaelis-Menten Kinetics

Figure 2(b) shows the uni-uni enzyme-catalyzed reaction scheme including inhibition. Figure 2(a) depicts an example of one possible corresponding CellDesigner process diagram. In the case of the

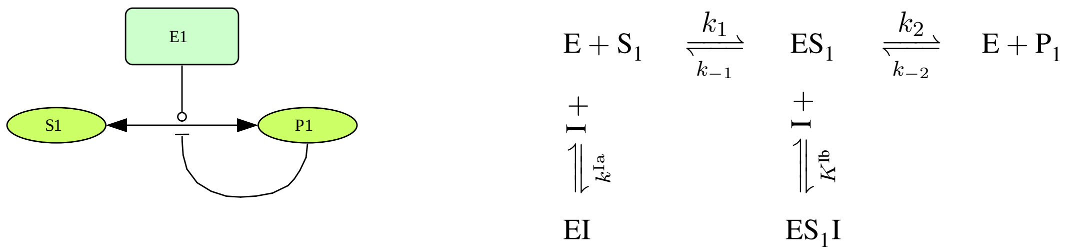

(a) Example of a process diagram

(b) Reaction scheme

Figure 2: Uni-uni enzyme-catalyzed reaction

enzyme-catalyzed uni-uni reaction, SBMLsqueezer can use convenience kinetics or, by default, the Michaelis-Menten equation. The latter includes both constants k^{Ia} and K^{Ib}. This allows for optimization of the model to fit the parameters and to decide which kind of inhibition is the most appropriate one if it is not known, including the following three special cases, that are often of particular interest:

1. Competitive inhibition for $0 < k^{\mathrm{Ia}} < \infty$ and $K^{\mathrm{Ib}} \to \infty$
2. Noncompetitive inhibition for $0 < k^{\mathrm{Ia}} = K^{\mathrm{Ib}} < \infty$
3. Uncompetitive inhibition for $k^{\mathrm{Ia}} \to \infty$ and $0 < K^{\mathrm{Ib}} < \infty$

A detailed explanation of the different kinds of modification can be found in “The Regulation of Cellular Systems” [11]. In addition to the well-described inhibition, we employ the activation prefactor of Equation (1).

The general Michaelis-Menten equation is given in Equation (9) with its corresponding irreversible form in Equation (10) and the example generated for the process diagram in Figure 2(a) is written in

[1]The zeroth order irreversible mass action kinetics, discrete scheme corresponds to the continuous form SBO:0000047.

[2]The first order irreversible mass action kinetics, discrete scheme corresponds to the continuous form SBO:0000049.

[3]The second order irreversible mass action kinetics, two reactants, discrete scheme corresponds to the continuous form SBO:0000054.

[4]The third order irreversible mass action kinetics, three reactants, discrete form corresponds to the continuous form SBO:0000061.

Equation (11).

$$v_j(\mathbf{S},\mathbf{p}) = [\mathrm{E}]_0 \cdot \prod_m h^{\mathrm{A}}([\mathrm{S}_m]k^{\mathrm{A}}_{jm})^{w^+_{jm}} \cdot \frac{\frac{k^{\mathrm{cat}}_{+j}}{k^{\mathrm{M}}_{j,\mathrm{S}_1}}[\mathrm{S}_1] - \frac{k^{\mathrm{cat}}_{-j}}{k^{\mathrm{M}}_{j,\mathrm{P}_1}}[\mathrm{P}_1]}{1 + \frac{[\mathrm{I}]}{k^{\mathrm{Ia}}_j} + \left(\frac{[\mathrm{S}_1]}{k^{\mathrm{M}}_{j,\mathrm{S}_1}} + \frac{[\mathrm{P}_1]}{k^{\mathrm{M}}_{j,\mathrm{P}_1}}\right)\left(1 + \frac{[\mathrm{I}]}{K^{\mathrm{Ib}}_j}\right)} \tag{9}$$

$$v_j(\mathbf{S},\mathbf{p}) = [\mathrm{E}]_0 \cdot \prod_m h^{\mathrm{A}}([\mathrm{S}_m]k^{\mathrm{A}}_{jm})^{w^+_{jm}} \cdot \frac{k^{\mathrm{cat}}_{+j}[\mathrm{S}_1]}{k^{\mathrm{M}}_1 + \frac{k^{\mathrm{M}}_{j,\mathrm{S}_1}}{k^{\mathrm{Ia}}_j}[\mathrm{I}] + [\mathrm{S}_1] + \frac{k^{\mathrm{M}}_{j,\mathrm{S}_1}}{K^{\mathrm{Ib}}_j}[\mathrm{I}]} \tag{10}$$

$$v_1 = [\mathrm{E}_1] \cdot \frac{\frac{k^{\mathrm{cat}}_{+1}}{k^{\mathrm{M}}_{1,\mathrm{S}_1}}[\mathrm{S}_1] - \frac{k^{\mathrm{cat}}_{-1}}{k^{\mathrm{M}}_{1,\mathrm{P}_1}}[\mathrm{P}\ 1]}{1 + \frac{[\mathrm{P}_1]}{k^{\mathrm{Ia}}_1} + \left(\frac{[\mathrm{S}_1]}{k^{\mathrm{M}}_{1,\mathrm{S}_1}} + \frac{[\mathrm{P}_1]}{k^{\mathrm{M}}_{1,\mathrm{P}_1}}\right)\left(1 + \frac{[\mathrm{P}_1]}{K^{\mathrm{Ib}}_1}\right)} \tag{11}$$

The SBO defines several special cases of Equation (10) and provides one special case of Equation (9) without inhibition (SBO:0000326). Activation is currently not considered in SBO. In the case of no modulation, Equation (10) covers SBO:0000028 to SBO:0000031 and SBO:0000199. If exactly one inhibitor interferes with the reaction, Equation (10) equals SBO:0000265. If $k^{\mathrm{Ia}}_j = K^{\mathrm{Ib}}_j$ Equation (10) and SBO:0000266 are identical. This equation also covers competitive inhibition with appropriate parameter settings: SBO:0000262 for $k^{\mathrm{Ia}}_j \to \infty$ and SBO:0000260 for $K^{\mathrm{Ib}}_j \to \infty$.

If more than one inhibitor interacts with the enzyme during the irreversible uni-uni reaction, SBMLsqueezer applies the mixed-type inhibition of irreversible enzymes by mutually exclusive inhibitors (SBO:0000275):

$$v_j(\mathbf{S},\mathbf{p}) = \frac{k^{\mathrm{cat}}_{+j}[\mathrm{E}]_0[\mathrm{S}_1]}{k^{\mathrm{M}}_{j,\mathrm{S}_1} \cdot \left(1 + \sum_{i=1}^{n} \frac{[\mathrm{I}_i]}{K^{\mathrm{Ib}i}_j}\right) + [\mathrm{S}_1] \cdot \left(1 + \sum_{i=1}^{n} \frac{[\mathrm{I}_i]}{K^{\mathrm{Ia}i}_j}\right)}. \tag{12}$$

This equation includes SBO:0000276 and SBO:0000277 if exactly two inhibitors lower the reaction velocity v_j. The latter one applies when $\forall i : K^{\mathrm{Ia}i} = K^{\mathrm{Ib}i}$, which depends on the parameter settings. Another special case of Equation (12) emerges for $\forall i : K^{\mathrm{Ia}i}_j \to \infty$: this rate law then includes SBO:0000270 (competitive inhibition of irreversible unireactant enzymes by exclusive inhibition) and its derivatives SBO:0000271 and SBO:0000274 (Table 2).

The competitive inhibition of irreversible unireactant enzymes by non-exclusive non-cooperative inhibitors (SBO:0000273) and its derivative SBO:0000267 constitute a special case of Equation (10) only if exactly one inhibitor interferes with the reaction, $K^{\mathrm{Ib}}_j \to \infty$ and the exponent $m_i = 1$:

$$v_j(\mathbf{S},\mathbf{p}) = \prod_m h^{\mathrm{A}}([\mathrm{S}_m]k^{\mathrm{A}}_{jm})^{w^+_{jm}} \cdot [\mathrm{E}]_0 \cdot \frac{k^{\mathrm{cat}}_j[\mathrm{S}_1]}{k^{\mathrm{M}}_{j,\mathrm{S}_1} \cdot \prod_{i=1}^{n}\left(1 + \frac{[\mathrm{I}_i]}{K^{\mathrm{I}}_i}\right)^{m_i} + [\mathrm{S}_1]}. \tag{13}$$

Therefore, SBMLsqueezer offers this equation as an alternative for each irreversible reaction with one substrate molecule and more than one inhibitor. Activation is included using the prefactor from convenience kinetics.

Reversible uni-uni reactions with more than one inhibitor are modeled using the following equation which makes use of Equation (1) and is not included in the SBO:

$$v_j(\mathbf{S},\mathbf{p}) = [\mathrm{E}]_0 \cdot f_j(\mathbf{S},\mathbf{p}) \cdot \frac{\frac{k^{\mathrm{cat}}_{+j}}{k^{\mathrm{M}}_{j,\mathrm{S}_1}}[\mathrm{S}_1] - \frac{k^{\mathrm{cat}}_{-j}}{k^{\mathrm{M}}_{j,\mathrm{P}_1}}[\mathrm{P}_1]}{1 + \frac{[\mathrm{S}_1]}{k^{\mathrm{M}}_{j,\mathrm{S}_1}} + \frac{[\mathrm{P}_1]}{k^{\mathrm{M}}_{j,\mathrm{P}_1}}}. \tag{14}$$

2.3. Bi-Uni Enzyme Reactions

In some cases a single enzyme reacts with two reactants. Depending on the sequence in which the reactants bind to the enzyme, we can distinguish two different reaction mechanisms. Additionally, convenience kinetics constitutes a third alternative when no information about the mechanism is available. For irreversible bi-uni enzyme reactions without modulation, Equation (32) gives an additional modeling alternative. A special case of this bi-uni reaction emerges if there is one reactant species that has a stoichiometric coefficient of two. Figure 3(a) shows a possible graphical representation of this type of reaction. Neither the random order mechanism nor the ordered mechanism for bi-uni reactions is currently defined in SBO. For both mechanisms we also apply the prefactor defined by convenience kinetics in Equation (1).

2.3.1. Random Order Mechanism

The reaction scheme of this mechanism is presented in Figure 3(b). For the sake of simplicity the inhibition mechanism is omitted from this scheme. Both substrates bind in arbitrary sequence to the enzyme. The general formula for this mechanism is given in Equation (15), and its irreversible form

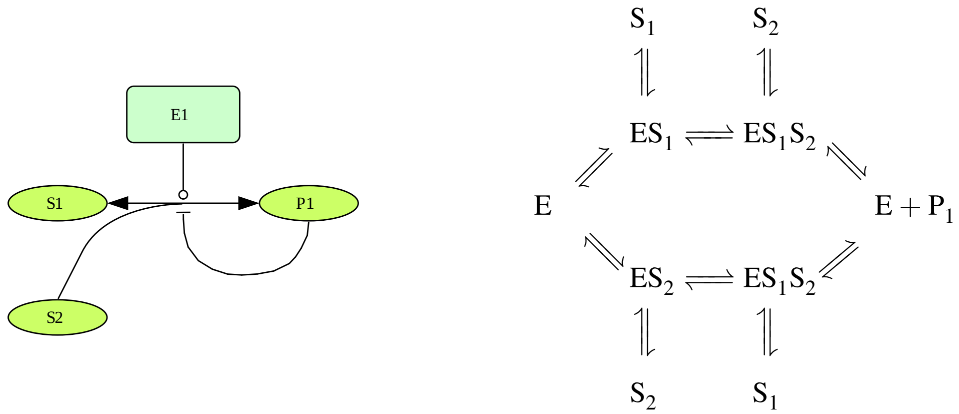

(a) Bi-uni enzyme-catalyzed reaction (b) Reaction scheme

Figure 3: Bi-uni random order enzyme reaction mechanism

is shown in Equation (16). The automatically generated equation to Figure 3(a) with respect to this mechanism can be found in Equation (17). For a derivation of this formula see Section 3.2 (page 22).

$$v_j(\mathbf{S},\mathbf{p}) = f_j(\mathbf{S},\mathbf{p}) \cdot \frac{\frac{k^{\mathrm{cat}}_{+j}[\mathrm{E}]_0[\mathrm{S}_1][\mathrm{S}_2]}{k^{\mathrm{I}}_{j,\mathrm{S}_1}k^{\mathrm{M}}_{j,\mathrm{S}_2}} - \frac{k^{\mathrm{cat}}_{-j}[\mathrm{E}]_0[\mathrm{P}_1]}{k^{\mathrm{M}}_{j,\mathrm{P}_1}}}{1 + \frac{[\mathrm{S}_1]}{k^{\mathrm{I}}_{j,\mathrm{S}_1}} + \frac{[\mathrm{S}_2]}{k^{\mathrm{I}}_{j,\mathrm{S}_2}} + \frac{[\mathrm{S}_1][\mathrm{S}_2]}{k^{\mathrm{M}}_{j,\mathrm{S}_2}k^{\mathrm{I}}_{j,\mathrm{S}_1}} + \frac{[\mathrm{P}_1]}{k^{\mathrm{M}}_{j,\mathrm{P}_1}}} \tag{15}$$

$$v_j(\mathbf{S},\mathbf{p}) = f_j(\mathbf{S},\mathbf{p}) \cdot \frac{k^{\mathrm{cat}}_{+j}[\mathrm{E}]_0[\mathrm{S}_1][\mathrm{S}_2]}{k^{\mathrm{I}}_{j,\mathrm{S}_1}k^{\mathrm{M}}_{j,\mathrm{S}_2} + k^{\mathrm{M}}_{j,\mathrm{S}_2}[\mathrm{S}_1] + k^{\mathrm{M}}_{j,\mathrm{S}_1}[\mathrm{S}_2] + [\mathrm{S}_1][\mathrm{S}_2]} \tag{16}$$

$$v_1 = \frac{k^{\mathrm{I}}_{1,\mathrm{P}_1}}{k^{\mathrm{I}}_{1,\mathrm{P}_1} + [\mathrm{P}_1]} \cdot \frac{\frac{k^{\mathrm{cat}}_{+1}}{k^{\mathrm{i}}_{1,\mathrm{S}_1}k^{\mathrm{M}}_{1,\mathrm{S}_2}}[\mathrm{E}_1][\mathrm{S}_1][\mathrm{S}_2] - \frac{k^{\mathrm{cat}}_{-1}}{k^{\mathrm{M}}_{1,\mathrm{P}_1}}[\mathrm{E}_1][\mathrm{P}_1]}{1 + \frac{[\mathrm{S}_1]}{k^{\mathrm{i}}_{1,\mathrm{S}_1}} + \frac{[\mathrm{S}_2]}{k^{\mathrm{i}}_{1,\mathrm{S}_2}} + \frac{[\mathrm{S}_1][\mathrm{S}_2]}{k^{\mathrm{i}}_{1,\mathrm{S}_1}k^{\mathrm{M}}_{1,\mathrm{S}_2}} + \frac{[\mathrm{P}_1]}{k^{\mathrm{M}}_{1,\mathrm{P}_1}}} \tag{17}$$

2.3.2. Ordered Mechanism

Figure 4(b) depicts the reaction scheme of the ordered bi-uni mechanism. Note that in this reaction mechanism the sequence in which the species react is fixed. Equation (18) gives the general formula

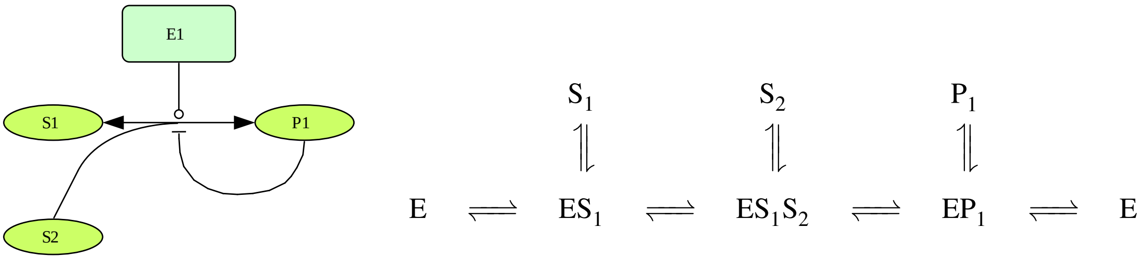

(a) Bi-uni enzyme-catalyzed reaction (b) Reaction scheme

Figure 4: Reaction scheme of the ordered bi-uni mechanism

of this mechanism and Equation (19) shows its corresponding irreversible version. Equation (20) was automatically generated with respect to Figure 4(a).

$$v_j(\mathbf{S},\mathbf{p}) = f_j(\mathbf{S},\mathbf{p}) \cdot \frac{\frac{k^{\text{cat}}_{+j}[\text{E}]_0[\text{S}_1][\text{S}_2]}{k^{\text{I}}_{j,\text{S}_1} k^{\text{M}}_{j,\text{S}_2}} - \frac{k^{\text{cat}}_{-j}[\text{E}]_0[\text{P}_1]}{k^{\text{M}}_{j,\text{P}_1}}}{1 + \frac{[\text{S}_1]}{k^{\text{I}}_{j,\text{S}_1}} + \frac{k^{\text{M}}_{j,\text{S}_1}[\text{S}_2]}{k^{\text{I}}_{j,\text{S}_1} k^{\text{M}}_{j,\text{S}_2}} + \frac{[\text{S}_1][\text{S}_2]}{k^{\text{M}}_{j,\text{S}_2} k^{\text{I}}_{j,\text{S}_1}} + \frac{k^{\text{M}}_{j,\text{S}_1}[\text{S}_2][\text{P}_1]}{k^{\text{I}}_{j,\text{S}_1} k^{\text{M}}_{j,\text{S}_2} k^{\text{I}}_{j,\text{P}_1}} + \frac{[\text{P}_1]}{k^{\text{M}}_{j,\text{P}_1}}} \tag{18}$$

$$v_j(\mathbf{S},\mathbf{p}) = f_j(\mathbf{S},\mathbf{p}) \cdot \frac{k^{\text{cat}}_{+j}[\text{E}]_0[\text{S}_1][\text{S}_2]}{k^{\text{I}}_{j,\text{S}_1} k^{\text{M}}_{j,\text{S}_2} + k^{\text{M}}_{j,\text{S}_2}[\text{S}_1] + k^{\text{M}}_{j,\text{S}_1}[\text{S}_2] + [\text{S}_1][\text{S}_2]} \tag{19}$$

$$v_1 = \frac{k^{\text{I}}_{1,\text{P}_1}}{k^{\text{I}}_{1,\text{P}_1} + [\text{P}_1]} \cdot \frac{\frac{k^{\text{cat}}_{+1}}{k^{\text{i}}_{1,\text{S}_1} k^{\text{M}}_{1,\text{S}_2}}[\text{E}_1][\text{S}_1][\text{S}_2] - \frac{k^{\text{cat}}_{-1}}{k^{\text{M}}_{1,\text{P}_1}}[\text{E}_1][\text{P}_1]}{1 + \frac{[\text{S}_1]}{k^{\text{i}}_{1,\text{S}_1}} + \frac{k^{\text{M}}_{1,\text{S}_1}[\text{S}\ 2]}{k^{\text{i}}_{1,\text{S}_1} k^{\text{M}}_{1,\text{S}_2}} + \frac{[\text{S}_1][\text{S}_2]}{k^{\text{i}}_{1,\text{S}_1} k^{\text{M}}_{1,\text{S}_2}} + \frac{k^{\text{M}}_{1,\text{S}_1}[\text{S}_2][\text{P}_1]}{k^{\text{i}}_{1,\text{S}_1} k^{\text{M}}_{1,\text{S}_2} k^{\text{i}}_{1,\text{P}_1}} + \frac{[\text{P}_1]}{k^{\text{M}}_{1,\text{P}_1}}} \tag{20}$$

For the derivation of this formula see Section 3.1 (page 18).

2.4. Bi-Bi Enzyme Reactions

Another special case covered by SBMLsqueezer is that of enzyme-catalyzed reactions with two substrates and two products. As was the case for the bi-uni reactions we can observe the possible mechanisms random order, ordered and, if no information about the mechanism is available, convenience kinetics. Additionally, a fourth reaction scheme can be applied: the bi-bi ping-pong mechanism, that also has a fixed sequence in which all participating molecules bind to the enzyme. Furthermore, irreversible reactions without modulation can also be described by Equation (32). Figure 5 illustrates one example of a process diagram for bi-bi reactions. As in the case of bi-uni reactions, it is also possible that only one substrate with stoichiometry coefficient two occurs. Here, there might also be just one product with stoichiometry coefficient two. In all following reaction schemes the mechanisms for inhibition were omitted due to the fact that modulation is included according to Equation (1). At the time of writing, the random order, ordered and ping-pong mechanisms for bi-bi reactions presented in the following sections were not defined by SBO.

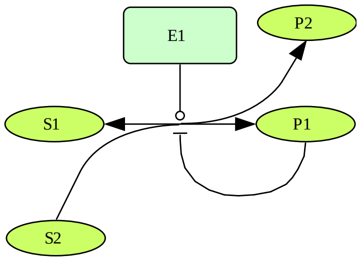

Figure 5: An example of a bi-bi enzyme reaction

2.4.1. Random Order Mechanism

The general reaction scheme of the random order mechanism for bi-bi reactions is given in Figure 6. The sequence in which the reactants bind to the enzyme and the products leave the enzyme complex is arbitrary. Equation (21) models the reversible reaction with this rapid-equilibrium random order

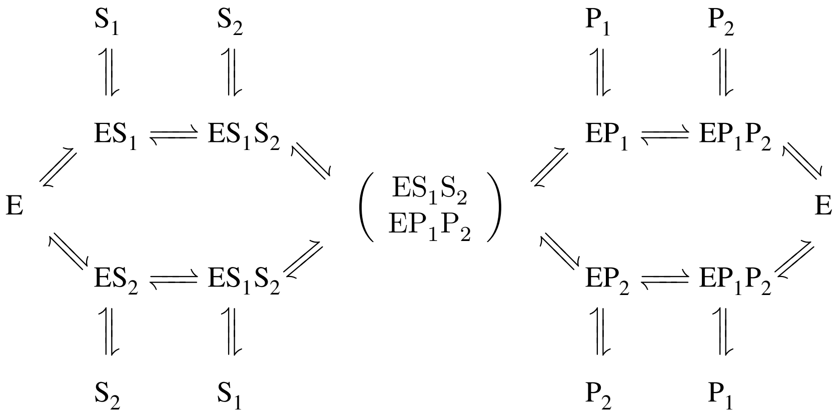

Figure 6: Reaction scheme of the random order bi-bi mechanism

ternary-complex mechanism [2, p. 169] whereas the irreversible alternative is given by Equation (22). The automatically derived equation for the example in Figure 5 is shown in Equation (23).

$$v_j(\mathbf{S},\mathbf{p}) = f_j(\mathbf{S},\mathbf{p}) \cdot \frac{\frac{k^{\mathrm{cat}}_{+j}[\mathrm{E}]_0[\mathrm{S}_1][\mathrm{S}_2]}{k^{\mathrm{I}}_{j,\mathrm{S}_1}k^{\mathrm{M}}_{j,\mathrm{S}_2}} - \frac{k^{\mathrm{cat}}_{-j}[\mathrm{E}]_0[\mathrm{P}_1][\mathrm{P}_2]}{k^{\mathrm{M}}_{j,\mathrm{P}_1}k^{\mathrm{I}}_{j,\mathrm{P}_2}}}{1 + \frac{[\mathrm{S}_1]}{k^{\mathrm{I}}_{j,\mathrm{S}_1}} + \frac{[\mathrm{S}_2]}{k^{\mathrm{I}}_{j,\mathrm{S}_2}} + \frac{[\mathrm{S}_1][\mathrm{S}_2]}{k^{\mathrm{M}}_{j,\mathrm{S}_2}k^{\mathrm{I}}_{j,\mathrm{S}_1}} + \frac{[\mathrm{P}_1]}{k^{\mathrm{I}}_{j,\mathrm{P}_1}} + \frac{[\mathrm{P}_2]}{k^{\mathrm{I}}_{j,\mathrm{P}_2}} + \frac{[\mathrm{P}_1][\mathrm{P}_2]}{k^{\mathrm{I}}_{j,\mathrm{P}_2}k^{\mathrm{M}}_{j,\mathrm{P}_1}}} \tag{21}$$

$$v_j(\mathbf{S},\mathbf{p}) = f_j(\mathbf{S},\mathbf{p}) \cdot \frac{k^{\mathrm{cat}}_{+j}[\mathrm{E}]_0[\mathrm{S}_1][\mathrm{S}_2]}{k^{\mathrm{I}}_{j,\mathrm{S}_1}k^{\mathrm{M}}_{j,\mathrm{S}_2} + k^{\mathrm{M}}_{j,\mathrm{S}_2}[\mathrm{S}_1] + k^{\mathrm{M}}_{j,\mathrm{S}_1}[\mathrm{S}_2] + [\mathrm{S}_1][\mathrm{S}_2]} \tag{22}$$

$$v_1 = \frac{k^{\mathrm{I}}_{1,\mathrm{P}_1}}{k^{\mathrm{I}}_{1,\mathrm{P}_1} + [\mathrm{P}_1]} \cdot \frac{\frac{k^{\mathrm{cat}}_{+1}}{k^{\mathrm{i}}_{1,\mathrm{S}_1}k^{\mathrm{M}}_{1,\mathrm{S}_2}}[\mathrm{E}_1][\mathrm{S}_1][\mathrm{S}_2] - \frac{k^{\mathrm{cat}}_{-1}}{k^{\mathrm{i}}_{1,\mathrm{P}_2}k^{\mathrm{M}}_{1,\mathrm{P}_1}}[\mathrm{E}_1][\mathrm{P}_1][\mathrm{P}_2]}{1 + \frac{[\mathrm{S}_1]}{k^{\mathrm{i}}_{1,\mathrm{S}_1}} + \frac{[\mathrm{S}_2]}{k^{\mathrm{i}}_{1,\mathrm{S}_2}} + \frac{[\mathrm{P}_1]}{k^{\mathrm{i}}_{1,\mathrm{P}_1}} + \frac{[\mathrm{P}_2]}{k^{\mathrm{i}}_{1,\mathrm{P}_2}} + \frac{[\mathrm{P}_1][\mathrm{P}_2]}{k^{\mathrm{i}}_{1,\mathrm{P}_2}k^{\mathrm{M}}_{1,\mathrm{P}_1}} + \frac{[\mathrm{S}_1][\mathrm{S}_2]}{k^{\mathrm{i}}_{1,\mathrm{S}_1}k^{\mathrm{M}}_{1,\mathrm{S}_2}}} \tag{23}$$

Due to the following relations among the Michaelis and inhibition constants, the constants $k^{\mathrm{M}}_{j,\mathrm{S}_1}$ and $k^{\mathrm{M}}_{j,\mathrm{P}_2}$ do not appear explicitly in Equation (21):

$$k^{\mathrm{M}}_{j,\mathrm{S}_1} k^{\mathrm{I}}_{j,\mathrm{S}_2} = k^{\mathrm{I}}_{j,\mathrm{S}_1} k^{\mathrm{M}}_{j,\mathrm{S}_2} \quad (24)$$

$$k^{\mathrm{M}}_{j,\mathrm{P}_1} k^{\mathrm{I}}_{j,\mathrm{S}_2} = k^{\mathrm{I}}_{j,\mathrm{P}_1} k^{\mathrm{M}}_{j,\mathrm{P}_2}. \quad (25)$$

2.4.2. Ordered Mechanism

Figure 7(b) presents the reaction scheme for the ordered bi-bi mechanism, which is also called the compulsory-order ternary-complex mechanism [2, pp. 166-168]. As in the bi-uni case (Section 2.3.2), the sequence, in which all reactants bind to the enzyme, is fixed. Furthermore, the products also leave the enzyme complex in a defined sequence. A special case of this reaction is given when there is just one reactant or just one product with the stoichiometry of two.

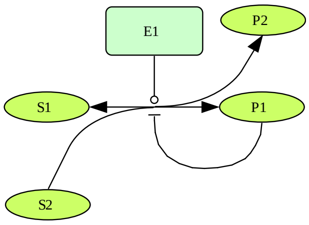

(a) Example of a bi-bi enzyme reaction

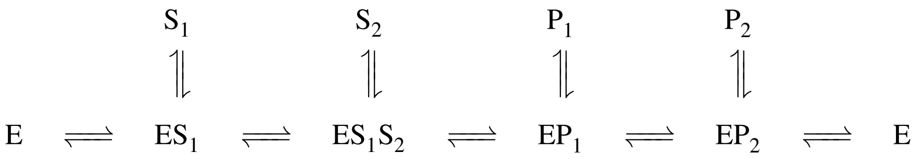

(b) Reaction scheme

Figure 7: Reaction scheme for the ordered bi-bi mechanism

The formula for a reversible reaction is given by Equation (26) whereas the corresponding irreversible form can be found in Equation (27). An example for a generated equation with respect to Figure 5 can be found in Equation (28).

$$
\begin{aligned}
v_j(\mathbf{S},\mathbf{p}) =& f_j(\mathbf{S},\mathbf{p}) \\
&\cdot \frac{\frac{k^{\mathrm{cat}}_{+j}[\mathrm{E}]_0[\mathrm{S}_1][\mathrm{S}_2]}{k^{\mathrm{I}}_{j,\mathrm{S}_1}k^{\mathrm{M}}_{j,\mathrm{S}_2}} - \frac{k^{\mathrm{cat}}_{-j}[\mathrm{E}]_0[\mathrm{P}_1][\mathrm{P}_2]}{k^{\mathrm{M}}_{j,\mathrm{P}_1}k^{\mathrm{I}}_{j,\mathrm{P}_2}}}{1+\frac{[\mathrm{S}_1]}{k^{\mathrm{I}}_{j,\mathrm{S}_1}}+\frac{k^{\mathrm{M}}_{j,\mathrm{S}_1}[\mathrm{S}_2]}{k^{\mathrm{I}}_{j,\mathrm{S}_1}k^{\mathrm{M}}_{j,\mathrm{S}_2}}+\frac{[\mathrm{S}_1][\mathrm{S}_2]}{k^{\mathrm{M}}_{j,\mathrm{S}_2}k^{\mathrm{I}}_{j,\mathrm{S}_1}}+\frac{k^{\mathrm{M}}_{j,\mathrm{S}_1}[\mathrm{S}_2][\mathrm{P}_1]}{k^{\mathrm{I}}_{j,\mathrm{S}_1}k^{\mathrm{M}}_{j,\mathrm{S}_2}k^{\mathrm{I}}_{j,\mathrm{P}_1}}+\frac{[\mathrm{P}_2]}{k^{\mathrm{I}}_{j,\mathrm{P}_2}}+\frac{k^{\mathrm{M}}_{j,\mathrm{P}_2}[\mathrm{P}_1]}{k^{\mathrm{I}}_{j,\mathrm{P}_2}k^{\mathrm{M}}_{j,\mathrm{P}_1}}+\frac{k^{\mathrm{M}}_{j,\mathrm{P}_2}[\mathrm{S}_1][\mathrm{P}_1]}{k^{\mathrm{I}}_{j,\mathrm{S}_1}k^{\mathrm{M}}_{j,\mathrm{P}_1}k^{\mathrm{I}}_{j,\mathrm{P}_2}}+\frac{[\mathrm{P}_1][\mathrm{P}_2]}{k^{\mathrm{I}}_{j,\mathrm{P}_2}k^{\mathrm{M}}_{j,\mathrm{P}_1}}+\frac{[\mathrm{S}_1][\mathrm{S}_2][\mathrm{P}_1]}{k^{\mathrm{I}}_{j,\mathrm{S}_1}k^{\mathrm{M}}_{j,\mathrm{S}_2}k^{\mathrm{I}}_{j,\mathrm{P}_1}}+\frac{[\mathrm{S}_2][\mathrm{P}_1][\mathrm{P}_2]}{k^{\mathrm{I}}_{j,\mathrm{S}_2}k^{\mathrm{M}}_{j,\mathrm{P}_1}k^{\mathrm{I}}_{j,\mathrm{P}_2}}}
\end{aligned}
\tag{26}
$$

$$
v_j(\mathbf{S},\mathbf{p}) = f_j(\mathbf{S},\mathbf{p}) \cdot \frac{k^{\mathrm{cat}}_{+j}[\mathrm{E}]_0[\mathrm{S}_1][\mathrm{S}_2]}{k^{\mathrm{I}}_{j,\mathrm{S}_1}k^{\mathrm{M}}_{j,\mathrm{S}_2}+k^{\mathrm{M}}_{j,\mathrm{S}_2}[\mathrm{S}_1]+k^{\mathrm{M}}_{j,\mathrm{S}_1}[\mathrm{S}_2]+[\mathrm{S}_1][\mathrm{S}_2]}
\tag{27}
$$

$$
\begin{aligned}
v_1 =& \frac{k^{\mathrm{I}}_{1,\mathrm{P}_1}}{k^{\mathrm{I}}_{1,\mathrm{P}_1}+[\mathrm{P}_1]} \\
&\cdot \frac{\frac{k^{\mathrm{cat}}_{+1}}{k^{\mathrm{i}}_{1,\mathrm{S}_1}k^{\mathrm{M}}_{1,\mathrm{S}_2}}[\mathrm{E}_1][\mathrm{S}_1][\mathrm{S}_2] - \frac{k^{\mathrm{cat}}_{-1}}{k^{\mathrm{i}}_{1,\mathrm{P}_2}k^{\mathrm{M}}_{1,\mathrm{P}_1}}[\mathrm{E}_1][\mathrm{P}_1][\mathrm{P}_2]}{1+\frac{[\mathrm{S}_1]}{k^{\mathrm{i}}_{1,\mathrm{S}_1}}+\frac{k^{\mathrm{M}}_{1,\mathrm{S}_1}[\mathrm{S}_2]}{k^{\mathrm{i}}_{1,\mathrm{S}_1}k^{\mathrm{M}}_{1,\mathrm{S}_2}}+\frac{k^{\mathrm{M}}_{1,\mathrm{P}_2}[\mathrm{P}_1]}{k^{\mathrm{i}}_{1,\mathrm{P}_2}k^{\mathrm{M}}_{1,\mathrm{P}_1}}+\frac{[\mathrm{P}_2]}{k^{\mathrm{i}}_{1,\mathrm{P}_2}}+\frac{[\mathrm{S}_1][\mathrm{S}_2]}{k^{\mathrm{i}}_{1,\mathrm{S}_1}k^{\mathrm{M}}_{1,\mathrm{S}_2}}+\frac{k^{\mathrm{M}}_{1,\mathrm{P}_2}[\mathrm{S}_1][\mathrm{P}_1]}{k^{\mathrm{i}}_{1,\mathrm{S}_1}k^{\mathrm{M}}_{1,\mathrm{P}_1}k^{\mathrm{i}}_{1,\mathrm{P}_2}}+\frac{k^{\mathrm{M}}_{1,\mathrm{S}_1}[\mathrm{S}_2][\mathrm{P}_2]}{k^{\mathrm{i}}_{1,\mathrm{S}_1}k^{\mathrm{M}}_{1,\mathrm{S}_2}k^{\mathrm{i}}_{1,\mathrm{P}_2}}+\frac{[\mathrm{P}_1][\mathrm{P}_2]}{k^{\mathrm{M}}_{1,\mathrm{P}_1}k^{\mathrm{i}}_{1,\mathrm{P}_2}}+\frac{[\mathrm{S}_1][\mathrm{S}_2][\mathrm{P}_1]}{k^{\mathrm{i}}_{1,\mathrm{S}_1}k^{\mathrm{M}}_{1,\mathrm{S}_2}k^{\mathrm{i}}_{1,\mathrm{P}_1}}+\frac{[\mathrm{S}_2][\mathrm{P}_1][\mathrm{P}_2]}{k^{\mathrm{i}}_{1,\mathrm{S}_2}k^{\mathrm{M}}_{1,\mathrm{P}_1}k^{\mathrm{i}}_{1,\mathrm{P}_2}}}
\end{aligned}
\tag{28}
$$

2.4.3. Ping-Pong Mechanism

A special case of the ordered mechanism is the ping-pong reaction, whose scheme is presented in Figure 8. The reactants bind in a fixed sequence, and the products leave the enzyme complex in a specific succession. However, during the reaction, the enzyme passes through different states so that it can only react with the next reactant or set the next product free. This is why the mechanism is also called a substituted-enzyme mechanism. No corresponding bi-uni reaction exists because it would formally be equal to the ordered bi-uni mechanism. Equation (29) gives the general formula

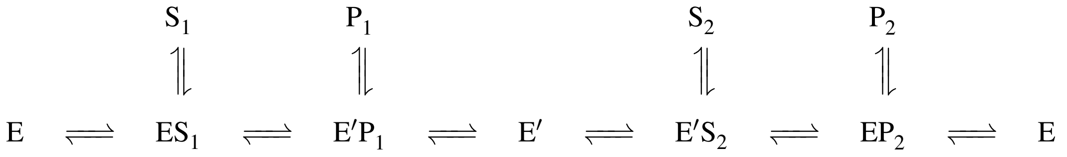

Figure 8: Reaction scheme of the ping-pong bi-bi mechanism

for this particular mechanism, and its corresponding irreversible form is shown in Equation (30) [2, pp. 169-171]. Equation (31) was generated by SBMLsqueezer with respect to the example in Figure 5.

$$v_j(\mathbf{S},\mathbf{p}) = f_j(\mathbf{S},\mathbf{p}) \cdot \frac{\frac{k^{\mathrm{cat}}_{+j}[\mathrm{E}]_0[\mathrm{S}_1][\mathrm{S}_2]}{k^{\mathrm{I}}_{j,\mathrm{S}_1}k^{\mathrm{M}}_{j,\mathrm{S}_2}} - \frac{k^{\mathrm{cat}}_{-j}[\mathrm{E}]_0[\mathrm{P}_1][\mathrm{P}_2]}{k^{\mathrm{M}}_{j,\mathrm{P}_2}k^{\mathrm{I}}_{j,\mathrm{P}_1}}}{\frac{[\mathrm{S}_1]}{k^{\mathrm{I}}_{j,\mathrm{S}_1}} + \frac{k^{\mathrm{M}}_{j,\mathrm{S}_1}[\mathrm{S}_2]}{k^{\mathrm{I}}_{j,\mathrm{S}_1}k^{\mathrm{M}}_{j,\mathrm{S}_2}} + \frac{[\mathrm{S}_1][\mathrm{S}_2]}{k^{\mathrm{M}}_{j,\mathrm{S}_2}k^{\mathrm{I}}_{j,\mathrm{S}_1}} + \frac{k^{\mathrm{M}}_{j,\mathrm{S}_1}[\mathrm{S}_2][\mathrm{P}_2]}{k^{\mathrm{I}}_{j,\mathrm{S}_1}k^{\mathrm{M}}_{j,\mathrm{S}_2}k^{\mathrm{I}}_{j,\mathrm{P}_2}} + \frac{[\mathrm{P}_1]}{k^{\mathrm{I}}_{j,\mathrm{P}_1}} + \frac{k^{\mathrm{M}}_{j,\mathrm{P}_1}[\mathrm{P}_2]}{k^{\mathrm{I}}_{j,\mathrm{P}_1}k^{\mathrm{M}}_{j,\mathrm{P}_2}} + \frac{[\mathrm{S}_1][\mathrm{P}_1]}{k^{\mathrm{I}}_{j,\mathrm{S}_1}k^{\mathrm{I}}_{j,\mathrm{P}_1}} + \frac{[\mathrm{P}_1][\mathrm{P}_2]}{k^{\mathrm{I}}_{j,\mathrm{P}_1}k^{\mathrm{M}}_{j,\mathrm{P}_2}}} \tag{29}$$

$$v_j(\mathbf{S},\mathbf{p}) = f_j(\mathbf{S},\mathbf{p}) \cdot \frac{k^{\mathrm{cat}}_{+j}[\mathrm{E}]_0[\mathrm{S}_1][\mathrm{S}_2]}{k^{\mathrm{M}}_{j,\mathrm{S}_2}[\mathrm{S}_1] + k^{\mathrm{M}}_{j,\mathrm{S}_1}[\mathrm{S}_2] + [\mathrm{S}_1][\mathrm{S}_2]} \tag{30}$$

$$v_1 = \frac{k^{\mathrm{I}}_{1,\mathrm{P}_1}}{k^{\mathrm{I}}_{1,\mathrm{P}_1} + [\mathrm{P}_1]} \cdot \frac{\frac{k^{\mathrm{cat}}_{+1}}{k^{\mathrm{i}}_{1,\mathrm{S}_1}k^{\mathrm{M}}_{1,\mathrm{S}_2}}[\mathrm{E}_1][\mathrm{S}_1][\mathrm{S}_2] - \frac{k^{\mathrm{cat}}_{-1}}{k^{\mathrm{i}}_{1,\mathrm{P}_1}k^{\mathrm{M}}_{1,\mathrm{P}_2}}[\mathrm{E}_1][\mathrm{P}_1][\mathrm{P}_2]}{\frac{[\mathrm{S}_1]}{k^{\mathrm{i}}_{1,\mathrm{S}_1}} + \frac{k^{\mathrm{M}}_{1,\mathrm{S}_1}[\mathrm{S}_2]}{k^{\mathrm{i}}_{1,\mathrm{S}_1}k^{\mathrm{M}}_{1,\mathrm{S}_2}} + \frac{[\mathrm{P}_1]}{k^{\mathrm{i}}_{1,\mathrm{P}_1}} + \frac{k^{\mathrm{M}}_{1,\mathrm{P}_1}[\mathrm{P}_2]}{k^{\mathrm{i}}_{1,\mathrm{P}_1}k^{\mathrm{M}}_{1,\mathrm{P}_2}} + \frac{[\mathrm{S}_1][\mathrm{S}_2]}{k^{\mathrm{i}}_{1,\mathrm{S}_1}k^{\mathrm{M}}_{1,\mathrm{S}_2}} + \frac{[\mathrm{S}_1][\mathrm{P}_1]}{k^{\mathrm{i}}_{1,\mathrm{S}_1}k^{\mathrm{i}}_{1,\mathrm{P}_1}} + \frac{k^{\mathrm{M}}_{1,\mathrm{S}_1}[\mathrm{S}_2][\mathrm{P}_2]}{k^{\mathrm{i}}_{1,\mathrm{S}_1}k^{\mathrm{M}}_{1,\mathrm{S}_2}k^{\mathrm{i}}_{1,\mathrm{P}_2}} + \frac{[\mathrm{P}_1][\mathrm{P}_2]}{k^{\mathrm{i}}_{1,\mathrm{P}_1}k^{\mathrm{M}}_{1,\mathrm{P}_2}}} \tag{31}$$

Comparing all bi-bi kinetic formulas, one can see that the ordered mechanism is the slowest one because of the large denominator.

2.5. Irreversible Non-Modulated Non-Interacting Reactant Enzymes

Irreversible enzyme-catalyzed reactions with more than one substrate can alternatively be modeled using the following equation if there is no modulator:

$$v_j(\mathbf{S},\mathbf{p}) = [E]_0 k_{+j} \prod_{i=1}^{n} \frac{[\mathrm{S}_i]}{k^{\mathrm{M}}_{ji} + [\mathrm{S}_i]}. \tag{32}$$

In contrast to the formulas for reversible reactions, the number of products does not matter for rate laws of irreversible reactions. Figure 9 depicts an example of a compatible process diagram. Two

substrate molecules react to one product. Equation (33) gives the generated rate law for this example.

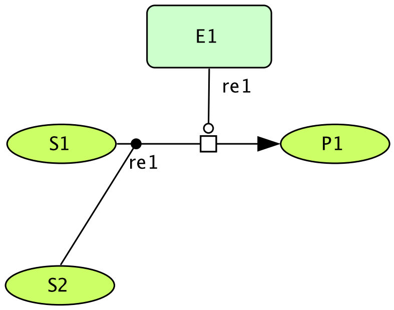

Figure 9: Example of an irreversible non-modulated non-interacting bireactant enzyme-catalyzed reaction

$$v_1 = \frac{k_1^{\text{cat}} \cdot [\text{E}_1] \cdot \frac{[\text{S}_1]}{k^{\text{M}}_{1,\text{S}_2}} \cdot \frac{[\text{S}_1]}{k^{\text{M}}_{1,\text{S}_1}}}{\left(1 + \frac{[\text{S}_2]}{k^{\text{M}}_{1,\text{S}_2}}\right) \cdot \left(1 + \frac{[\text{S}_1]}{k^{\text{M}}_{1,\text{S}_1}}\right)} \tag{33}$$

SBMLsqueezer offers the user the choice of selecting this equation (SBO:0000150) whenever the aforementioned conditions are fulfilled. Equation (32) also covers the special cases SBO:0000151 and SBO:0000152 for two or three substrate molecules, respectively. This rate law is a special case of convenience kinetics with distinct reactants, each with stoichiometry one and no modulation at all.

2.6. Convenience Kinetics and Thermodynamics

In their original work Liebermeister *et al.* published the convenience kinetics in two forms [4]:

$$v_j(\mathbf{S}, \mathbf{p}) = f_j(\mathbf{S}, \mathbf{p}) \cdot [\text{E}_j] \cdot \frac{k^{\text{cat}}_{+j} \prod_i \left(\frac{[\text{S}_i]}{K^{\text{M}}_{ji}}\right)^{n^-_{ij}} - k^{\text{cat}}_{-j} \prod_i \left(\frac{[\text{S}_i]}{K^{\text{M}}_{ji}}\right)^{n^+_{ij}}}{\prod_i \sum_{m=0}^{n^-_{ij}} \left(\frac{[\text{S}_i]}{K^{\text{M}}_{ji}}\right)^m + \prod_i \sum_{m=0}^{n^+_{ij}} \left(\frac{[\text{S}_i]}{K^{\text{M}}_{ji}}\right)^m - 1} \tag{34}$$

$$v_j(\mathbf{S}, \mathbf{p}) = f_j(\mathbf{S}, \mathbf{p}) \cdot k^{\text{V}}_j \cdot [\text{E}_j] \cdot \frac{\prod_i \left(\frac{[\text{S}_i]}{K^{\text{M}}_{ji}}\right)^{n^-_{ij}} \left(k^{\text{G}}_i k^{\text{M}}_{ji}\right)^{-\frac{n_{ij}}{2}} - \prod_i \left(\frac{[\text{S}_i]}{K^{\text{M}}_{ji}}\right)^{n^+_{ij}} \left(k^{\text{G}}_i k^{\text{M}}_{ji}\right)^{\frac{n_{ij}}{2}}}{\prod_i \sum_{m=0}^{n^-_{ij}} \left(\frac{[\text{S}_i]}{K^{\text{M}}_{ji}}\right)^m + \prod_i \sum_{m=0}^{n^+_{ij}} \left(\frac{[\text{S}_i]}{K^{\text{M}}_{ji}}\right)^m - 1}. \tag{35}$$

The prefactor f introduces the modifiers for activation or inhibition, to the kinetic equation and was defined in Equation (1).

Equation (34) can be applied to any enzyme-catalyzed reaction. However, if the stoichiometric matrix $\mathbf{N}$ of the reaction system contains linearly dependent columns, i. e., $\mathbf{N}$ does not have full column rank, then at least one reaction is thermodynamically dependent on another. In this case, choosing the parameters of the equation while ignoring this dependency may fit given measurement data well but will violate the thermodynamic constraints of the system. Hence, Liebermeister *et al.* derived a second form of convenience kinetics, which is shown in Equation (35). The parameters $k^{\text{cat}}_{\pm j}$ are replaced by $\prod_i \left(k^{\text{G}}_i k^{\text{M}}_{ji}\right)^{\mp\frac{n_{ij}}{2}}$ and the whole fraction is multiplied by the additional parameter

k_j^{V}. This ensures that all newly introduced parameters are thermodynamically independent. Note that every k_i^{G} stands for molecule i regardless of the respective reaction, whereas every k_j^{V} is a parameter for reaction j and does not depend on any molecule. The Michaelis analog parameter k_{ji}^{M} depends on both reaction j and molecule i and thus links both parameters together. For a complete derivation see the original paper of Liebermeister *et al.* [4].

Because Equation (35) is more complicated and contains additional parameters, SBMLsqueezer uses the simpler formula whenever applicable. To ensure the thermodynamic correctness of the system, an implementation of the Gaussian algorithm, which computes the rank of a matrix, is invoked. If the stoichiometric matrix of the system has full column rank, there is no need to apply Equation (35). Otherwise SBMLsqueezer will assign every reaction to be modeled using convenience kinetics with Equation (35).

For all enzyme-catalyzed reactions independent of the mechanism, convenience kinetics may be an appropriate choice if the user lacks detailed biochemical knowledge. As stated before, reactions with more than two substrate molecules are unlikely to take place. SBMLsqueezer will show a warning message for such reactions. This number does not only stand for the number of different reactant species, but rather for the stoichiometry on the left hand side. For instance, the reaction

$$4\,\mathrm{A} \longrightarrow 6\,\mathrm{B} \tag{36}$$

will also be considered unrealistic. This warning is, however, user-defined and the equations can still be generated properly pursuant to the particular formula. In the case of the context menu, warnings are shown whenever the number of reacting species exceeds two.

In an application of convenience kinetics to a mixed network together with uni-uni Michaelis-Menten equations, it was shown that convenience kinetics leads to reasonable results when fitted to *in vivo* data [12]. At the time of writing no form of convenience kinetics is included in the systems biology ontology (SBO, Table 2).

2.6.1. Thermodynamically Dependent Form

Equation (34) shows the thermodynamically dependent formula of convenience kinetics for reversible reactions. Equation (37) gives its corresponding irreversible form [4]. An example of a generated rate law is shown in Equation (38) for the bi-bi reaction presented in Figure 5 on page 11.

$$v_j(\mathbf{S},\mathbf{p}) = f_j(\mathbf{S},\mathbf{p}) \cdot [\mathrm{E}]_0 \frac{k_{+j}^{\mathrm{cat}} \prod_i \left(\frac{[\mathrm{S}_i]}{k_{ji}^{\mathrm{M}}}\right)^{n_{ij}^{-}}}{\prod_i \sum_{m=0}^{n_{il}^{-}} \left(\frac{[\mathrm{S}_i]}{k_{ji}^{\mathrm{M}}}\right)^{m}} \tag{37}$$

$$v_1 = \frac{k_{1,\mathrm{P1}}^{\mathrm{I}}}{k_{1,\mathrm{P1}}^{\mathrm{I}} + [\mathrm{P}_1]} \cdot [\mathrm{E}_1] \cdot \frac{k_{+1}^{\mathrm{cat}} \cdot \frac{[\mathrm{S}_1]}{k_{1,\mathrm{S}_1}^{\mathrm{M}}} \cdot \frac{[\mathrm{S}_2]}{k_{1,\mathrm{S}_2}^{\mathrm{M}}} - k_{-1}^{\mathrm{cat}} \cdot \frac{[\mathrm{P}_1]}{k_{1,\mathrm{P}_1}^{\mathrm{M}}} \cdot \frac{[\mathrm{P}_2]}{k_{1,\mathrm{P}_2}^{\mathrm{M}}}}{\left(1 + \frac{[\mathrm{S}_1]}{k_{1,\mathrm{S}_1}^{\mathrm{M}}}\right)\left(1 + \frac{[\mathrm{S}_2]}{k_{1,\mathrm{S}_2}^{\mathrm{M}}}\right) + \left(1 + \frac{[\mathrm{P}_1]}{k_{1,\mathrm{P}_1}^{\mathrm{M}}}\right)\left(1 + \frac{[\mathrm{P}_2]}{k_{1,\mathrm{P}_2}^{\mathrm{M}}}\right) - 1} \tag{38}$$

2.6.2. Thermodynamically Independent Form

As stated at the beginning of this Section (page 15), if there are linear dependencies within the stoichiometric matrix, SBMLsqueezer applies the thermodynamically independent form of convenience kinetics, which is shown in Equation (35) and for its corresponding irreversible form in Equation (39)

[4]. Equation (40) shows the generated independent form of the reaction example depicted in Figure 5, page 11.

$$v_j(\mathbf{S},\mathbf{p}) = f_j(\mathbf{S},\mathbf{p}) \cdot [\mathrm{E}] \cdot k_l^{\mathrm{V}} \frac{\prod_i \left(\frac{[\mathrm{S}_i]}{k_{ji}^{\mathrm{M}}}\right)^{n_{il}^{-}} \left(k_i^{\mathrm{G}} k_{ji}^{\mathrm{M}}\right)^{-\frac{n_{ij}}{2}}}{\prod_i \sum_{m=0}^{n_{il}^{-}} \left(\frac{[\mathrm{S}_i]}{k_{ji}^{\mathrm{M}}}\right)^{m}} \tag{39}$$

$$v_1 = \frac{k_{1,\mathrm{P1}}^{\mathrm{I}}}{k_{1,\mathrm{P1}}^{\mathrm{I}} + [\mathrm{P}_1]} \cdot [\mathrm{E}_1] \cdot k_1^{\mathrm{V}} \cdot \frac{\frac{[\mathrm{S}_1]}{k_{1,\mathrm{S}_1}^{\mathrm{M}}} \cdot \frac{[\mathrm{S}_2]}{k_{1,\mathrm{S}_2}^{\mathrm{M}}} \sqrt{\frac{k_{\mathrm{S1}}^{\mathrm{G}} k_{1,\mathrm{S}_1}^{\mathrm{M}} k_{\mathrm{S2}}^{\mathrm{G}} k_{1,\mathrm{S}_2}^{\mathrm{M}}}{k_{\mathrm{P1}}^{\mathrm{G}} k_{1,\mathrm{P}_1}^{\mathrm{M}} k_{\mathrm{P2}}^{\mathrm{G}} k_{1,\mathrm{P}_2}^{\mathrm{M}}}} - \frac{[\mathrm{P}_1]}{k_{1,\mathrm{P}_1}^{\mathrm{M}}} \cdot \frac{[\mathrm{P}_2]}{k_{1,\mathrm{P}_2}^{\mathrm{M}}} \sqrt{\frac{k_{\mathrm{P1}}^{\mathrm{G}} k_{1,\mathrm{P}_1}^{\mathrm{M}} k_{\mathrm{P2}}^{\mathrm{G}} k_{1,\mathrm{P}_2}^{\mathrm{M}}}{k_{\mathrm{S1}}^{\mathrm{G}} k_{1,\mathrm{S}_1}^{\mathrm{M}} k_{\mathrm{S2}}^{\mathrm{G}} k_{1,\mathrm{S}_2}^{\mathrm{M}}}}}{\left(1 + \frac{[\mathrm{S}_1]}{k_{1,\mathrm{S}_1}^{\mathrm{M}}}\right)\left(1 + \frac{[\mathrm{S}_2]}{k_{1,\mathrm{S}_2}^{\mathrm{M}}}\right) + \left(1 + \frac{[\mathrm{P}_1]}{k_{1,\mathrm{P}_1}^{\mathrm{M}}}\right)\left(1 + \frac{[\mathrm{P}_2]}{k_{1,\mathrm{P}_2}^{\mathrm{M}}}\right) - 1} \tag{40}$$

2.7. Hill Equation

Gene regulation can also be modeled using CellDesigner. A common rate law to model those reactions is the Hill equation [11, 13]. Figure 10 depicts one example of a process considered gene regulation. Gene s_1 is expressed and the RNA molecule s_2 assembles. This process is (transcriptionally) inhibited by the translation product, protein s_3. The translation process is (translationally) activated by protein s_4. Note that the concentration of gene s_1 remains unchanged during this process as the transcription does not change the state of the gene. SBMLsqueezer recognizes mistakes within the SBML file and sets the boundary conditions of genes to "true". Furthermore, SBMLsqueezer will show warnings if a transcription is, for instance, "translationally" activated. Since the release of CellDesigner 4.0β

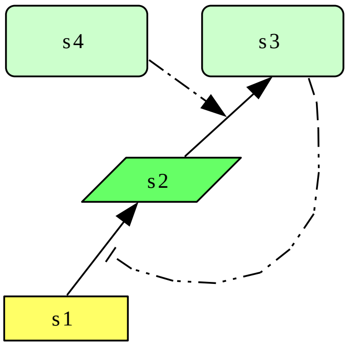

Figure 10: An example of gene expression regulation in CellDesigner notation

there have been two special arrows for the transitions described here: transcription and translation. To ensure backwards compability, SBMLsqueezer supports simple state transitions, even between genes and RNA as well as between RNA and proteins. However, if transcription and translation arrows are used, SBMLsqueezer will show a warning message if they are mixed up.

The general Hill equation is given in Equation (41). The formula for the translation example in Figure 10 can be found in Equation (42) and the rate of transcription generated according to Figure 10 is given by Equation (43). Note that the exponents $w_{jm}^{\pm}$ are defined according to the modulation

matrices $\mathbf{W}^{\pm}$ in Section 2.6, page 15.

$$v_j(\mathbf{S},\mathbf{p}) = v_j^{\mathrm{m}} \prod_m \left(\frac{[\mathrm{S}_m]^{n_{jm}^{\mathrm{H}}}}{[\mathrm{S}_m]^{n_{jm}^{\mathrm{H}}} + \left(K_{jm}^{\mathrm{S}}\right)^{n_{jm}^{\mathrm{H}}}} \right)^{w_{jm}^{+}} \left(1 - \frac{[\mathrm{S}_m]^{n_{jm}^{\mathrm{H}}}}{[\mathrm{S}_m]^{n_{jm}^{\mathrm{H}}} + \left(K_{jm}^{\mathrm{S}}\right)^{n_{jm}^{\mathrm{H}}}} \right)^{w_{jm}^{-}} \prod_i \mathrm{S}_i^{n_{ij}^{+} \cdot k_i} \tag{41}$$

$$v_1 = k_1^{\mathrm{g}} \cdot \left(1 - \frac{[\mathrm{s}_3]^{n_{-1,\mathrm{s}_3}}}{[\mathrm{s}_3]^{n_{-1,\mathrm{s}_3}} + \left(k_{-1,\mathrm{s}_3}^{\mathrm{S}}\right)^{n_{-1,\mathrm{s}_3}}} \right) \tag{42}$$

$$v_2 = k_2^{\mathrm{g}} \cdot \frac{[\mathrm{s}_4]^{n_{+2,\mathrm{s}_4}}}{[\mathrm{s}_4]^{n_{+2,\mathrm{s}_4}} + \left(k_{+2,\mathrm{s}_4}^{\mathrm{S}}\right)^{n_{+2,\mathrm{s}_4}}} \tag{43}$$

The constant k_i distinguishes genes from other species:

$$k_i = \begin{cases} 0 & \text{if } \mathrm{S_i} \text{ is a gene,} \\ 1 & \text{otherwise.} \end{cases} \tag{44}$$

The SBO defines three forms of the Hill equation (SBO:0000192, SBO:0000195 and SBO:0000198). The form described here is the general microscopic form (SBO:0000195) where no inhibition is involved; see Table 2 for details. Both other types are special cases of this formula for appropriate parameter settings. If a gene regulation or translation reaction without an assigned activator or inhibitor occurs, Equation (42) formally becomes a zeroth order mass-action equation (Section 2.1, page 6).

3. Derivation of Predefined Kinetic Equations for Bi-Uni Reactions

This section shows the derivation of rate laws for the random order and the ordered bi-uni mechanisms using the King-Altman method [2]. This derivation is necessary, since for this special case the common literature does not provide appropriate equations to be used as a pre-computed formula in SBMLsqueezer [2, 3, 14].

The King-Altman method provides an algorithm to create rate laws even for complex enzyme-catalyzed reaction mechanisms according to the quasi-steady-state approximation [2, 14]. The algorithm roughly consists of five steps that will be explained in detail in the remainder of this section.

3.1. Ordered Bi-Uni Mechanism

Firstly, we derive the rate law for the ordered bi-uni mechanism. Note that the sequence, in which the reactants bind to the enzyme molecule, is fixed (Figure 4(b), page 10).

First Step

A polygon, whose arcs and vertices reflect the reaction mechanism, is charted (Figure 11). Every vertex symbolizes one of the forms of the enzyme during the reaction. The edges mirror the transition between these forms. All charted transitions have to be first-order reactions. Second-order reactions must be given in pseudo-first-order form. The arrows, which constitute the edges of the diagram,

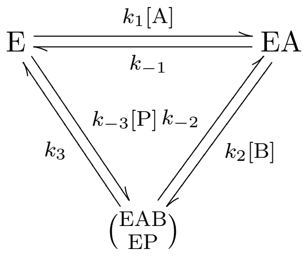

Figure 11: Underlying reaction scheme for the ordered bi-uni mechanism

are labeled with the rate constants, which are multiplied with entering ligands if necessary, for the corresponding transition.

The master pattern drafts the reaction scheme as a rough structure. In this case we obtain a triangle (Figure 12).

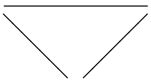

Figure 12: Master pattern of the ordered bi-uni mechanism

Second Step

Next, we construct all possible substructures of Figure 12 which

1. contain only edges of the master pattern
2. connect all enzyme states and
3. do not contain closed loops.

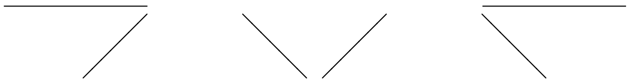

Figure 13: All valid sub-structures of the master pattern

Each of these patterns contains exactly one edge fewer than the master pattern. We obtain three structures, each with two edges (Figure 13).

Third Step

In this step every single enzyme state is marked one time within each pattern and directed arcs replace the edges, each pointing towards the highlighted enzyme state. According to the resulting pattern, we determine an equation for the relative amount of each highlighted enzyme state (Figure 14).

The denominator $\mathcal{D}$ equals the sum of all numerator terms of the equations in Figure 14 and reads

$$\mathcal{D} = k_{-1}k_{-2} + k_2k_3[\mathrm{B}] + k_{-1}k_3 + k_1k_2[\mathrm{A}][\mathrm{B}] + k_{-2}k_{-3}[\mathrm{B}][\mathrm{P}] + k_{-1}k_{-3}[\mathrm{P}] + k_1k_2[\mathrm{A}] + k_{-2}k_{-3}[\mathrm{P}] + k_1k_3[\mathrm{A}]. \quad (48)$$

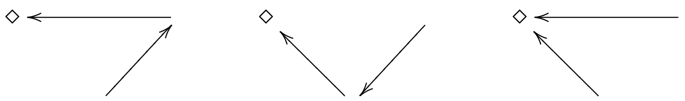

$$\frac{[\mathrm{E}]}{[\mathrm{E}]_0} = \frac{k_{-1}k_{-2} + k_2k_3[\mathrm{B}] + k_{-1}k_3}{\mathcal{D}} \tag{45}$$

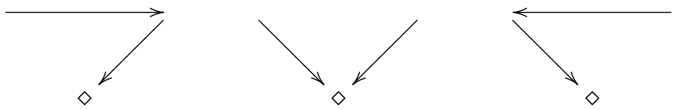

$$\frac{[\mathrm{EAB}]}{[\mathrm{E}]_0} = \frac{k_1k_2[\mathrm{A}][\mathrm{B}] + k_{-2}k_{-3}[\mathrm{B}][\mathrm{P}] + k_{-1}k_{-3}[\mathrm{P}]}{\mathcal{D}} \tag{46}$$

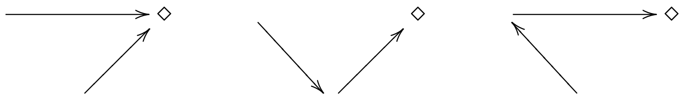

$$\frac{[\mathrm{EB}]}{[\mathrm{E}]_0} = \frac{k_1k_2[\mathrm{A}] + k_{-2}k_{-3}[\mathrm{P}] + k_1k_3[\mathrm{A}]}{\mathcal{D}} \tag{47}$$

Figure 14: Sub-patterns with their respective equations

Fourth Step

We now write the denominator as the product of coefficients in a way that all constants are ordered with respect to their concentration terms:

$$\mathcal{D} = \mathcal{D}_0 + [\mathrm{A}]\mathcal{D}_1 + [\mathrm{B}]\mathcal{D}_2 + [\mathrm{A}][\mathrm{B}]\mathcal{D}_3 + [\mathrm{B}][\mathrm{P}]\mathcal{D}_4 + [\mathrm{P}]\mathcal{D}_5 \tag{49}$$

where

$$\mathcal{D}_0 = k_{-1}(k_{-2} + k_3) \tag{50}$$
$$\mathcal{D}_1 = k_1(k_{-2} + k_3) \tag{51}$$
$$\mathcal{D}_2 = k_2 + k_3 \tag{52}$$
$$\mathcal{D}_3 = k_1 + k_2 \tag{53}$$
$$\mathcal{D}_4 = k_2 + k_{-3} \tag{54}$$
$$\mathcal{D}_5 = k_{-3}(k_{-1} + k_{-2}). \tag{55}$$

The rate law is then given as the sum of the rates to form a particular product decremented by the rates that reduce this product. In this ordered bi-uni mechanism there is only one step, in which P is

produced. Hence, there is only one way to consume P again and the formula reads:

$$v = \frac{\mathrm{d[P]}}{\mathrm{d}t} = k_3[\mathrm{EAB}] - k_{-3}[\mathrm{E}][\mathrm{P}] \tag{56}$$

$$\begin{aligned} &= [\mathrm{E}]_0 \frac{k_1k_2k_3[\mathrm{A}][\mathrm{B}] + k_2k_3k_{-3}[\mathrm{B}][\mathrm{P}] + k_{-1}k_3k_{-3}[\mathrm{P}]}{\mathcal{D}} \\ &+ \frac{-k_{-1}k_{-2}k_{-3}[\mathrm{P}] - k_2k_3k_{-3}[\mathrm{B}][\mathrm{P}] - k_{-1}k_3k_{-3}[\mathrm{P}]}{\mathcal{D}} \\ &= [\mathrm{E}]_0 \frac{k_1k_2k_3[\mathrm{A}][\mathrm{B}] - k_{-1}k_{-2}k_{-3}[\mathrm{P}]}{\mathcal{D}}. \end{aligned} \tag{57}$$

Fifth Step

The kinetic parameters are defined based on the coefficients determined in the fourth step. The Michaelis constants k_i^{M} are defined as the ratio of all constants of the substrate or product formation rate, minus the constants of the product or substrate formation rate, and the coefficient of the rates of all substrates or products.

$$k_{\mathrm{A}}^{\mathrm{M}} = \frac{\mathcal{D}_2}{\mathcal{D}_3} = \frac{k_2k_3}{k_1k_2} = \frac{k_3}{k_1} \tag{58}$$

$$k_{\mathrm{B}}^{\mathrm{M}} = \frac{\mathcal{D}_1}{\mathcal{D}_3} = \frac{k_1(k_{-2}+k_3)}{k_1k_2} = \frac{k_{-2}+k_3}{k_2} \tag{59}$$

$$k_{\mathrm{P}}^{\mathrm{M}} = \frac{\mathcal{D}_0}{\mathcal{D}_5} = \frac{k_{-1}(k_{-2}+k_3)}{k_{-3}(k_{-1}+K_{-2})} \tag{60}$$

The maximal activities for the forward and reverse reaction, V_+^{m} and V_-^{m}, are the quotient of the respective numerator coefficient and the coefficient of all substrates or products, respectively.

$$V_+^{\mathrm{m}} = \frac{\text{numerator}_1}{\text{coefficient of all substrates}} = [\mathrm{E}]_0 \frac{k_1k_2k_3}{k_1k_2} \tag{61}$$

$$V_-^{\mathrm{m}} = \frac{\text{numerator}_2}{\text{coefficient of all products}} = [\mathrm{E}]_0 \frac{k_{-1}k_{-2}k_{-3}}{k_{-3}(k_{-1}+k_{-2})} \tag{62}$$

After some conversions these kinetic equations can be combined as follows:

$$[\mathrm{E}]_0 k_{-1}k_{-2}k_{-3} = V_+^{\mathrm{m}} k_1k_2 \tag{63}$$

and

$$[\mathrm{E}][\mathrm{E}]_0 k_1k_2k_3 = V_-^{\mathrm{m}} k_{-3}(k_{-1}+k_{-2}). \tag{64}$$

Applying some more conversions and aggregation, the rate law then reads

$$v = \frac{V_+^{\mathrm{m}}k_1k_2[\mathrm{A}][\mathrm{B}] - V_-^{\mathrm{m}}k_{-3}(k_{-1}+k_{-2})[\mathrm{P}]}{\mathcal{D}} \tag{65}$$

$$= \frac{\frac{V_+^{\mathrm{m}}k_1k_2[\mathrm{A}][\mathrm{B}] - V_-^{\mathrm{m}}k_{-3}(k_{-1}+k_{-2})[\mathrm{P}]}{k_{-1}(k_{-2}+k_3)}}{\frac{\mathcal{D}}{k_{-1}(k_{-2}+k_3)}} \tag{66}$$

$$= \frac{\frac{V_+^{\mathrm{m}}[\mathrm{A}][\mathrm{B}]}{k_{\mathrm{A}}^{\mathrm{I}}k_{\mathrm{B}}^{\mathrm{M}}} - \frac{V_-^{\mathrm{m}}[\mathrm{P}]}{k_{\mathrm{P}}^{\mathrm{M}}}}{1 + \frac{[\mathrm{A}]}{k_{\mathrm{A}}^{\mathrm{I}}} + \frac{k_{\mathrm{A}}^{\mathrm{M}}[\mathrm{B}]}{k_{\mathrm{A}}^{\mathrm{I}}k_{\mathrm{B}}^{\mathrm{M}}} + \frac{[\mathrm{A}][\mathrm{B}]}{k_{\mathrm{B}}^{\mathrm{M}}k_{\mathrm{A}}^{\mathrm{I}}} + \frac{k_{\mathrm{A}}^{\mathrm{M}}[\mathrm{B}][\mathrm{P}]}{k_{\mathrm{A}}^{\mathrm{I}}k_{\mathrm{B}}^{\mathrm{M}}k_{\mathrm{P}}^{\mathrm{I}}} + \frac{[\mathrm{P}]}{k_{\mathrm{P}}^{\mathrm{M}}}}, \tag{67}$$

where we define the following constants:

$$k_{\mathrm{P}}^{\mathrm{I}} = \frac{k_{-3}}{k_3} \tag{68}$$

$$k_{\mathrm{A}}^{\mathrm{I}} = \frac{k_{-1}}{k_1}. \tag{69}$$

To accommodate this equation with the uni-uni Michaelis-Menten equation, we set $V_{+}^{\mathrm{m}} = k_{+j}^{\mathrm{cat}}[\mathrm{E}]_0$ and $V_{-}^{\mathrm{m}} = k_{-j}^{\mathrm{cat}}[\mathrm{E}]_0$. This leads us to the following equation for a reversible ordered bi-uni mechanism:

$$v = \frac{\frac{k_{+j}^{\mathrm{cat}}[\mathrm{E}]_0[\mathrm{A}][\mathrm{B}]}{k_{\mathrm{A}}^{\mathrm{I}} k_{\mathrm{B}}^{\mathrm{M}}} - \frac{k_{-j}^{\mathrm{cat}}[\mathrm{E}]_0[\mathrm{P}]}{k_{\mathrm{P}}^{\mathrm{M}}}}{1 + \frac{[\mathrm{A}]}{k_{\mathrm{A}}^{\mathrm{I}}} + \frac{k_{\mathrm{A}}^{\mathrm{M}}[\mathrm{B}]}{k_{\mathrm{A}}^{\mathrm{I}} k_{\mathrm{B}}^{\mathrm{M}}} + \frac{[\mathrm{A}][\mathrm{B}]}{k_{\mathrm{B}}^{\mathrm{M}} k_{\mathrm{A}}^{\mathrm{I}}} + \frac{k_{\mathrm{A}}^{\mathrm{M}}[\mathrm{B}][\mathrm{P}]}{k_{\mathrm{A}}^{\mathrm{I}} k_{\mathrm{B}}^{\mathrm{M}} k_{\mathrm{P}}^{\mathrm{I}}} + \frac{[\mathrm{P}]}{k_{\mathrm{P}}^{\mathrm{M}}}}. \tag{70}$$

For the irreversible ordered bi-uni mechanism the formula for the rate law reads

$$v = \frac{\frac{k_{+j}^{\mathrm{cat}}[\mathrm{E}]_0[\mathrm{A}][\mathrm{B}]}{k_{\mathrm{A}}^{\mathrm{I}} k_{\mathrm{B}}^{\mathrm{M}}}}{1 + \frac{[\mathrm{A}]}{k_{\mathrm{A}}^{\mathrm{I}}} + \frac{k_{\mathrm{A}}^{\mathrm{M}}[\mathrm{B}]}{k_{\mathrm{A}}^{\mathrm{I}} k_{\mathrm{B}}^{\mathrm{M}}} + \frac{[\mathrm{A}][\mathrm{B}]}{k_{\mathrm{B}}^{\mathrm{M}} k_{\mathrm{A}}^{\mathrm{I}}}} = \frac{k_{+j}^{\mathrm{cat}}[\mathrm{E}]_0[\mathrm{A}][\mathrm{B}]}{k_{\mathrm{A}}^{\mathrm{I}} k_{\mathrm{B}}^{\mathrm{M}} + k_{\mathrm{B}}^{\mathrm{M}}[\mathrm{A}] + k_{\mathrm{A}}^{\mathrm{M}}[\mathrm{B}] + [\mathrm{A}][\mathrm{B}]}. \tag{71}$$

3.2. Random Order Bi-Uni Mechanism

The random order mechanism is characterized by its arbitrary sequence, in which the reactants bind to the enzyme. The binding of every substrate is carried out independently of the others (Figure 3(a) on page 9). This must also be reflected in the corresponding rate law.

To derive an appropriate pre-computed formula for this mechanism, we again apply the five steps of the King-Altman method [14, pp. 126-131]. Here, we only briefly summarize the steps and omit a detailed explanation.

First Step

We chart the master pattern of the mechanism from the polygon that belongs to this particular reaction scheme (Figure 15).

Second Step

Next we construct all possible substructures, each with exactly one edge fewer than the master pattern (Figure 16).

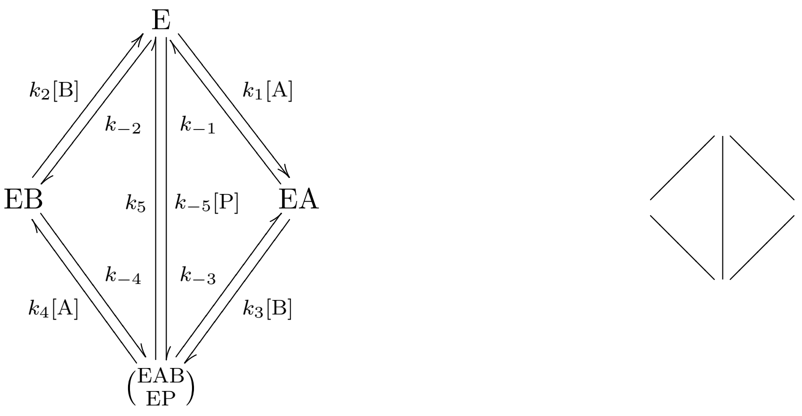

(a) Polygon showing the structure

(b) Master pattern

Figure 15: Structure and master pattern of the bi-uni random mechanism

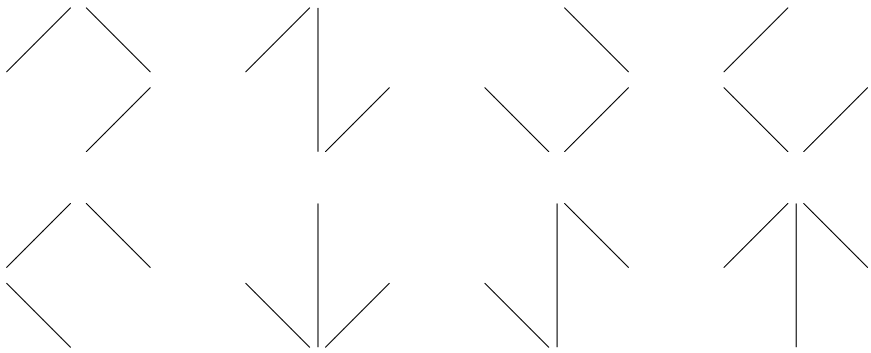

Figure 16: All valid sub-structures derived from the master pattern

Third Step

In this step, we derive the equations for every form of the enzyme attributable to the respective sub-patterns.

$$\frac{[\mathrm{E}]}{[\mathrm{E}]_0} = \frac{k_{-2}k_{-1}k_{-3} + k_{-1}k_{-3}k_4[\mathrm{A}] + k_{-2}k_{-4}k_3[\mathrm{B}] + k_{-4}k_{-2}k_{-1}}{\mathcal{D}} + \frac{k_5k_4k_3[\mathrm{A}][\mathrm{B}] + k_{-1}k_{-2}k_5 + k_{-2}k_3k_5[\mathrm{B}] + k_{-1}k_4k_5[\mathrm{A}]}{\mathcal{D}} \quad (72)$$

$$\frac{[\mathrm{E}][\mathrm{A}][\mathrm{B}]}{[\mathrm{E}]_0} = \frac{k_1k_{-2}k_3[\mathrm{A}][\mathrm{B}] + k_1k_3k_4[\mathrm{A}]^2[\mathrm{B}] + k_2k_3k_4[\mathrm{A}][\mathrm{B}]^2 + k_{-1}k_2k_4[\mathrm{A}][\mathrm{B}]}{\mathcal{D}} + \frac{k_3k_4k_{-5}[\mathrm{A}][\mathrm{B}][\mathrm{P}] + k_{-1}k_{-2}k_{-5}[\mathrm{P}] + k_{-2}k_3k_{-5}[\mathrm{B}][\mathrm{P}] + k_{-1}k_4k_{-5}[\mathrm{A}][\mathrm{P}]}{\mathcal{D}} \quad (73)$$

$$\frac{[\mathrm{E}][\mathrm{B}]}{[\mathrm{E}]_0} = \frac{k_{-1}k_2k_{-3}[\mathrm{B}] + k_1k_3k_{-4}[\mathrm{A}][\mathrm{B}] + k_2k_3k_{-4}[\mathrm{B}]^2 + k_{-1}k_2k_{-4}[\mathrm{B}]}{\mathcal{D}} + \frac{k_3k_{-4}k_{-5}[\mathrm{P}][\mathrm{B}] + k_{-1}k_2k_5[\mathrm{B}] + k_2k_3k_5[\mathrm{B}]^2 + k_{-1}k_{-4}k_{-5}[\mathrm{P}]}{\mathcal{D}} \tag{74}$$

$$\frac{[\mathrm{E}][\mathrm{A}]}{[\mathrm{E}]_0} = \frac{k_1k_{-2}k_{-3}[\mathrm{A}] + k_1k_{-3}k_4[\mathrm{A}]^2 + k_2k_{-3}k_4[\mathrm{A}][\mathrm{B}] + k_1k_{-2}k_{-4}[\mathrm{A}]}{\mathcal{D}} + \frac{k_{-3}k_4k_{-5}[\mathrm{A}][\mathrm{P}] + k_1k_{-2}k_5[\mathrm{A}] + k_{-2}k_{-3}k_{-5}[\mathrm{P}] + k_1k_4k_5[\mathrm{A}]^2}{\mathcal{D}} \tag{75}$$

Fourth Step

The denominator and the preliminary kinetic equation can now be written as:

$$\begin{aligned}\mathcal{D} =&(k_{-1}k_{-2}k_{-3} + k_{-1}k_{-2}k_{-4} + k_{-1}k_{-2}k_5)\\ &+ [\mathrm{A}](k_{-1}k_4k_5 + k_1k_{-2}k_{-3} + k_1k_{-2}k_{-4} + k_1k_{-2}k_5 + k_{-1}k_{-3}k_4)\\ &+ [\mathrm{A}]^2(k_1k_{-3}k_4 + k_1k_4k_5) + [\mathrm{A}][\mathrm{B}](k_3k_4k_5 + k_1k_{-2}k_3 + k_1k_3k_{-4} + k_2k_{-3}k_4 + k_{-1}k_2k_4)\\ &+ [\mathrm{A}]^2[\mathrm{B}](k_1k_3k_4) + [\mathrm{B}](k_{-2}k_3k_{-4} + k_{-2}k_3k_5 + k_{-1}k_2k_{-3} + k_{-1}k_2k_{-4} + k_{-1}k_2k_5)\\ &+ [\mathrm{B}]^2(k_2k_3k_{-4} + k_2k_3k_5) + [\mathrm{A}][\mathrm{B}]^2(k_2k_3k_4) + [\mathrm{P}](k_{-1}k_{-2}k_{-5} + k_{-1}k_{-4}k_{-5} + k_{-2}k_{-3}k_{-5})\\ &+ [\mathrm{A}][\mathrm{P}](k_{-3}k_4k_{-5} + k_{-1}k_4k_{-5}) + [\mathrm{A}][\mathrm{B}][\mathrm{P}](k_3k_4k_{-5}) + [\mathrm{B}][\mathrm{P}](k_3k_{-4}k_{-5} + k_{-2}k_3k_{-5})\\ =&\mathcal{D}_0 + [\mathrm{A}]\mathcal{D}_1 + [\mathrm{A}]^2\mathcal{D}_2 + [\mathrm{A}][\mathrm{B}]\mathcal{D}_3 + [\mathrm{A}]^2[\mathrm{B}]\mathcal{D}_4 + [\mathrm{B}]\mathcal{D}_5 + [\mathrm{B}]^2\mathcal{D}_6 +\\ &[\mathrm{A}][\mathrm{B}]^2\mathcal{D}_7 + [\mathrm{P}]\mathcal{D}_8 + [\mathrm{A}][\mathrm{P}]\mathcal{D}_9 + [\mathrm{A}][\mathrm{B}][\mathrm{P}]\mathcal{D}_{10} + [\mathrm{B}][\mathrm{P}]\mathcal{D}_{11}\end{aligned} \tag{76}$$

$$\begin{aligned}v = \frac{\mathrm{d}[\mathrm{P}]}{\mathrm{d}t} =& k_5[\mathrm{EAB}] - k_{-5}[\mathrm{E}][\mathrm{P}]\\ =&[\mathrm{E}]_0\frac{(k_1k_{-2}k_3 + k_{-1}k_2k_4)k_5[\mathrm{A}][\mathrm{B}] + k_1k_3k_4k_5[\mathrm{A}]^2[\mathrm{B}] + k_2k_3k_4k_5[\mathrm{A}][\mathrm{B}]^2}{\mathcal{D}}\\ &- \frac{(k_{-1}k_{-2} + k_{-1}k_{-2}k_{-3})k_{-5}[\mathrm{P}] + k_{-1}k_{-3}k_4k_{-3}[\mathrm{A}][\mathrm{P}] + k_{-2}k_3k_{-4}k_{-5}[\mathrm{B}][\mathrm{P}]}{\mathcal{D}}.\end{aligned} \tag{77}$$

Fifth Step

To complete the derivation, we have to define the Michaelis constant. According to Segel, the random order bi-uni mechanism does not provide a hyperbole function when no substrate saturation is present [3]. Thus, a kinetic formula based on this mechanism cannot be linearized. Hence, we may combine several rate constants to Michaelis-like constants similar to what we applied in the derivation for the ordered bi-uni mechanism. However, these constants would not be in accordance with the definition of the Michaelis constant of the uni-uni formula [3]. Assuming fast steady-states between the ternary and the binary complexes EAB and EP, and also assuming all conversions to be fast, this equation can be simplified as follows: According to Cornish-Bowden, the squared terms in the numerator and denominator as well as the terms [B][P], [A][P] and [A][B][P] then disappear [2]. This leads to the definition of the dissociation constants $k^{\mathrm{I}}_{\mathrm{A}}$, $k^{\mathrm{I}}_{\mathrm{B}}$ and $k^{\mathrm{I}}_{\mathrm{P}}$ for $k^{\mathrm{M}}_{\mathrm{A}}$, $k^{\mathrm{M}}_{\mathrm{B}}$ and $k^{\mathrm{M}}_{\mathrm{P}}$. Since the sequence, in which the reactants bind to the enzyme, is arbitrary, we have $k^{\mathrm{M}}_{\mathrm{A}}k^{\mathrm{I}}_{\mathrm{B}} = k^{\mathrm{I}}_{\mathrm{A}}k^{\mathrm{M}}_{\mathrm{B}}$. Hence, the derived equation is valid for this mechanism and assumes an underlying rapid-equilibrium-random-mechanism. The constants of this equation are defined as follows:

$$k^{\mathrm{M}}_{\mathrm{A}} = \frac{\mathcal{D}_5}{\mathcal{D}_3} \quad (78)$$

$$k^{\mathrm{M}}_{\mathrm{B}} = \frac{\mathcal{D}_1}{\mathcal{D}_3} \quad (79)$$

$$k^{\mathrm{M}}_{\mathrm{P}} = \frac{\mathcal{D}_0}{\mathcal{D}_8} \quad (80)$$

$$k^{\mathrm{I}}_{\mathrm{A}} = \frac{\mathcal{D}_1}{\mathcal{D}_0} \quad (81)$$

$$k^{\mathrm{I}}_{\mathrm{B}} = \frac{\mathcal{D}_0}{\mathcal{D}_5}. \quad (82)$$

And the final equation then reads:

$$v = \frac{\frac{k^{\mathrm{cat}}_{+j}[\mathrm{E}]_0[\mathrm{A}][\mathrm{B}]}{k^{\mathrm{I}}_{\mathrm{A}}k^{\mathrm{M}}_{\mathrm{B}}} - \frac{k^{\mathrm{cat}}_{-j}[\mathrm{E}]_0[\mathrm{P}]}{k^{\mathrm{M}}_{\mathrm{P}}}}{1 + \frac{[\mathrm{A}]}{k^{\mathrm{I}}_{\mathrm{A}}} + \frac{[\mathrm{B}]}{k^{\mathrm{I}}_{\mathrm{B}}} + \frac{[\mathrm{A}][\mathrm{B}]}{k^{\mathrm{M}}_{\mathrm{B}}k^{\mathrm{I}}_{\mathrm{A}}} + \frac{[\mathrm{P}]}{k^{\mathrm{M}}_{\mathrm{P}}}}. \quad (83)$$

For the irreversible random order bi-uni mechanism the above formula can be simplified to

$$v = \frac{\frac{k^{\mathrm{cat}}_{+j}[\mathrm{E}]_0[\mathrm{A}][\mathrm{B}]}{k^{\mathrm{I}}_{\mathrm{A}}k^{\mathrm{M}}_{\mathrm{B}}}}{1 + \frac{[\mathrm{A}]}{k^{\mathrm{I}}_{\mathrm{A}}} + \frac{[\mathrm{B}]}{k^{\mathrm{I}}_{\mathrm{B}}} + \frac{[\mathrm{A}][\mathrm{B}]}{k^{\mathrm{M}}_{\mathrm{B}}k^{\mathrm{I}}_{\mathrm{A}}}} = \frac{k^{\mathrm{cat}}_{+j}[\mathrm{E}]_0[\mathrm{A}][\mathrm{B}]}{k^{\mathrm{I}}_{\mathrm{A}}k^{\mathrm{M}}_{\mathrm{B}} + k^{\mathrm{M}}_{\mathrm{B}}[\mathrm{A}] + k^{\mathrm{M}}_{\mathrm{A}}[\mathrm{B}] + [\mathrm{A}][\mathrm{B}]}. \quad (84)$$

A. Systems Biology Ontology of Mathematical Expressions

The following Table 2 provides an overview of all mathematical expressions (SBO:0000064), defined mainly by Nicolas Le Novère, Michael Hucka and Andrew Finney in the Systems Biology Ontology [15]. The SBO term "mathematical expression" contains the term "conservation law" (SBO:0000355) and has two sub-categories: "rate law" (SBO:0000001) and "obsolete mathematical expression" (SBO:0000005).

Here we only consider the category "rate law", whose terms are listed in the following table. The identifiers of internal nodes are written in bold face. The column "variables" contains both parameters and reacting species. The table lists the tree of SBO terms for rate laws, which are defined at the time of writing, serially. The entries in this table are not sorted with respect to the SBO identification number but to the order of their occurrence within the tree from top to bottom. The discrete scheme mass-action kinetics, which are currently not supported by SBMLsqueezer, are printed gray. Note that four of these are formally equivalent to their corresponding continuous rate laws (see Section 2.1 for details).

Also, the mathematical form of the kinetics of irreversible non-modulated unireactant enzymes (SBO:0000028), the Henri-Michaelis-Menten equation (SBO:0000029), the Van Slyke and Cullen equation (SBO:0000030) as well as the Briggs-Haldane equation (SBO:0000031) are effectively identical. The lattermost one is almost the same as the other three except that the constant K^{S} is renamed to K^{M}. The difference in these equations can be explained by different modeling assumptions, in particular the denotation of K^{M}:

Michaelis-Menten, rapid equilibrium $K = \frac{k^{\mathrm{off}}}{k^{\mathrm{on}}}$

Briggs-Haldane, quasi-steady-state $K = \frac{k^{\mathrm{off}} + k^{\mathrm{cat}}}{k^{\mathrm{on}}}$

Van Slyke and Cullen, irreversible substrate binding $K = \frac{k^{\mathrm{cat}}}{k^{\mathrm{on}}}$

Table 2: The systems biology ontology (SBO) of rate laws

SBO	Term	Variables	Formula
**0000012**	Mass action kinetics	general category	
**0000041**	Irreversible mass action kinetics	general category	
**0000043**	Zeroth order irreversible mass action kinetics	general category	
**0000043**	First order irreversible mass action kinetics	general category	
**0000045**	Second order irreversible mass action kinetics	general category	
**0000050**	Second order irreversible mass action kinetics, one reactant	general category	
**0000053**	Second order irreversible mass action kinetics, two reactants	general category	
**0000055**	Third order irreversible mass action kinetics	general category	
**0000056**	Third order irreversible mass action kinetics, one reactant	general category	
**0000058**	Third order irreversible mass action kinetics, two reactants	general category	
**000060**	Third order irreversible mass action kinetics, three reactants	general category	
**0000163**	Irreversible mass action kinetics, continuous scheme	k, n, μ, R	$k \cdot \prod_{i=0}^{n} R_i^{\mu_i}$
0000047	Zeroth order irreversible mass action kinetics, continuous scheme	k	k
**0000049**	First order irreversible mass action kinetics, continuous scheme	k, R	$k \cdot R$
0000333	Monoexpotential decay	l, R	$\frac{R}{l}$
0000052	Second order irreversible mass action kinetics, one reactant, continuous scheme	k, R	$k \cdot R^2$
0000054	Second order irreversible mass action kinetics, two reactants, continuous scheme	k, R_1, R_2	$k \cdot R_1 \cdot R_2$
0000057	Third order irreversible mass action kinetics, one reactant, continuous scheme	k, R	$k \cdot R^2 \cdot R$

SBO	Term	Variables	Formula
0000059	Third order irreversible mass action kinetics, two reactants, continuous scheme	k, R_1, R_2	$k \cdot R_1^2 \cdot R_2$
0000061	Third order irreversible mass action kinetics, three reactants, continuous scheme	k, R_1, R_2, R_3	$k \cdot R_1 \cdot R_2 \cdot R_3$
**0000166**	Irreversible mass action kinetics, discrete scheme	c, n, μ, R	$c \cdot \prod_{i=0}^{n} \frac{R_i!}{(R_i-\mu_i)! \cdot \mu_i!}$
0000140	Zeroth order irreversible mass action kinetics, discrete scheme	c	c
0000141	First order irreversible mass action kinetics, discrete scheme	c, R	$c \cdot R$
0000142	Second order irreversible mass action kinetics, one reactant, discrete scheme	c, R	$c \cdot \frac{R \cdot (R-1)}{2}$
0000143	Second order irreversible mass action kinetics, two reactants, discrete scheme	c, R_1, R_2	$c \cdot R_1 \cdot R_2$
0000144	Third order irreversible mass action kinetics, one reactant, discrete scheme	c, R	$c \cdot \frac{R \cdot (R-1) \cdot (R-2)}{6}$
0000145	Third order irreversible mass action kinetics, two reactants, discrete scheme	c, R_1, R_2	$c \cdot R_1 \cdot \frac{R_2 \cdot (R_2-1)}{2}$
0000146	Third order irreversible mass action kinetics, three reactants, discrete scheme	c, R_1, R_2, R_3	$c \cdot R_1 \cdot R_2 \cdot R_3$
**0000042**	Reversible mass action kinetics	general category	
**0000069**	Zeroth order forward reversible mass action kinetics	general category	
0000070	Zeroth order forward, first order reverse, reversible mass action kinetics, continuous scheme	$k_\mathrm{f}, k_\mathrm{r}, P$	$k_\mathrm{f} - k_\mathrm{r} \cdot P$
**0000071**	Zeroth order forward, second order reverse, reversible mass action kinetics	general category	
0000072	Zeroth order forward, second order reverse with one product, reversible mass action kinetics, continuous scheme	$k_\mathrm{f}, k_\mathrm{r}, P$	$k_\mathrm{f} - k_\mathrm{r} \cdot P^2$

SBO	Term	Variables	Formula
0000073	Zeroth order forward, second order reverse with two products, reversible mass action kinetics, continuous scheme	k_f, k_r, P_1, P_2	$k_\mathrm{f} - k_\mathrm{r} \cdot P_1 \cdot P_2$
**0000074**	Zeroth order forward, third order reverse, reversible mass action kinetics	general category	
0000075	Zeroth order forward, third order reverse with one product, reversible mass action kinetics, continuous scheme	$k_\mathrm{f}, k_\mathrm{r}, P$	$k_\mathrm{f} - k_\mathrm{r} \cdot P^3$
0000076	Zeroth order forward, third order reverse with two products, reversible mass action kinetics, continuous scheme	k_f, k_r, P_1, P_2	$k_\mathrm{f} - k_\mathrm{r} \cdot P_1 \cdot P_2 \cdot P_2$
0000077	Zeroth order forward, third order reverse with three products, reversible mass action kinetics, continuous scheme	k_f, k_r, P_1, P_2, P_3	$k_\mathrm{f} - k_\mathrm{r} \cdot P_1 \cdot P_2 \cdot P_3$
**0000078**	First order forward reversible mass action kinetics	general category	
0000079	First order forward, zeroth order reverse, reversible mass action kinetics, continuous scheme	$k_\mathrm{f}, k_\mathrm{r}, R$	$k_\mathrm{f} \cdot R - k_\mathrm{r}$
0000080	First order forward, first order reverse, reversible mass action kinetics, continuous scheme	$k_\mathrm{f}, k_\mathrm{r}, R, P$	$k_\mathrm{f} \cdot R - k_\mathrm{r} \cdot P$
**0000081**	First order forward, second order reverse, reversible mass action kinetics	general category	
0000082	First order forward, second order reverse with one product, reversible mass action kinetics, continuous scheme	$k_\mathrm{f}, k_\mathrm{r}, R, P$	$k_\mathrm{f} \cdot R - k_\mathrm{r} \cdot P^2$
0000083	First order forward, second order reverse with two products, reversible mass action kinetics, continuous scheme	$k_\mathrm{f}, k_\mathrm{r}, R, P_1$, P_2	$k_\mathrm{f} \cdot R - k_\mathrm{r} \cdot P_1 \cdot P_2$
**0000084**	First order forward, third order reverse, reversible mass action kinetics	general category	

SBO	Term	Variables	Formula
0000085	First order forward, third order reverse with one product, reversible mass action kinetics, continuous scheme	$k_\mathrm{f}, k_\mathrm{r}, R, P$	$k_\mathrm{f} \cdot R - k_\mathrm{r} \cdot P^3$
0000086	First order forward, third order reverse with two products, reversible mass action kinetics, continuous scheme	$k_\mathrm{f}, k_\mathrm{r}, R, P_1$, P_2	$k_\mathrm{f} \cdot R - k_\mathrm{r} \cdot P_1^2 \cdot P_2$
0000087	First order forward, third order reverse with three products, reversible mass action kinetics, continuous scheme	$k_\mathrm{f}, k_\mathrm{r}, R, P_1$, P_2, P_3	$k_\mathrm{f} \cdot R - k_\mathrm{r} \cdot P_1 \cdot P_2 \cdot P_3$
**0000088**	Second order forward reversible mass action kinetics	general category	
**0000089**	Second order forward with one reactant reversible mass action kinetics	general category	
0000090	Second order forward with one reactant, zeroth order reverse, reversible mass action kinetics, continuous scheme	$k_\mathrm{f}, k_\mathrm{r}, R$	$k_\mathrm{f} \cdot R^2 - k_\mathrm{r}$
0000091	Second order forward with one reactant, first order reverse, reversible mass action kinetics, continuous scheme	$k_\mathrm{f}, k_\mathrm{r}, R, P$	$k_\mathrm{f} \cdot R^2 - k_\mathrm{r} \cdot P$
**0000092**	Second order forward with one reactant, second order reverse, reversible mass action kinetics	general category	
0000093	Second order forward with one reactant, second order reverse with one product, reversible mass action kinetics, continuous scheme	$k_\mathrm{f}, k_\mathrm{r}, R, P$	$k_\mathrm{f} \cdot R^2 - k_\mathrm{r} \cdot P^2$
0000094	Second order forward with one reactant, second order reverse with two products, reversible mass action kinetics, continuous scheme	$k_\mathrm{f}, k_\mathrm{r}, R, P_1$, P_2	$k_\mathrm{f} \cdot R^2 - k_\mathrm{r} \cdot P_1 \cdot P_2$
**0000095**	Second order forward with one reactant, third order reverse, reversible mass action kinetics	general category	

SBO	Term	Variables	Formula
0000096	Second order forward with one reactant, third order reverse with one product, reversible mass action kinetics, continuous scheme	$k_\mathrm{f}, k_\mathrm{r}, R, P$	$k_\mathrm{f} \cdot R^2 - k_\mathrm{r} \cdot P^3$
0000097	Second order forward with one reactant, third order reverse with two products, reversible mass action kinetics, continuous scheme	$k_\mathrm{f}, k_\mathrm{r}, R, P_1, P_2$	$k_\mathrm{f} \cdot R^2 - k_\mathrm{r} \cdot P_1^2 \cdot P_2$
0000098	Second order forward with one reactant, third order reverse with three products, reversible mass action kinetics, continuous scheme	$k_\mathrm{f}, k_\mathrm{r}, R, P_1, P_2, P_3$	$k_\mathrm{f} \cdot R^2 - k_\mathrm{r} \cdot P_1 \cdot P_2 \cdot P_3$
**0000099**	Second order forward with two reactants reversible mass action kinetics	general category	
0000100	Second order forward with two reactants, zeroth order reverse, reversible mass action kinetics, continuous scheme	$k_\mathrm{f}, k_\mathrm{r}, R_1, R_2$	$k_\mathrm{f} \cdot R_1 \cdot R_2 - k_\mathrm{r}$
0000101	Second order forward with two reactants, first order reverse, reversible mass action kinetics, continuous scheme	$k_\mathrm{f}, k_\mathrm{r}, R_1, R_2, P$	$k_\mathrm{f} \cdot R_1 \cdot R_2 - k_\mathrm{r} \cdot P$
**0000102**	Second order forward with two reactants, second order reverse, reversible mass action kinetics	general category	
0000103	Second order forward with two reactants, second order reverse with one product, reversible mass action kinetics, continuous scheme	$k_\mathrm{f}, k_\mathrm{r}, R_1, R_2, P$	$k_\mathrm{f} \cdot R_1 \cdot R_2 - k_\mathrm{r} \cdot P^2$
0000104	Second order forward with two reactants, second order reverse with two products, reversible mass action kinetics, continuous scheme	$k_\mathrm{f}, k_\mathrm{r}, R_1, R_2, P_1, P_2$	$k_\mathrm{f} \cdot R_1 \cdot R_2 - k_\mathrm{r} \cdot P_1 \cdot P_2$
**0000105**	Second order forward with two reactants, third order reverse, reversible mass action kinetics	general category	

SBO	Term	Variables	Formula
0000106	Second order forward with two reactants, third order reverse with one product, reversible mass action kinetics, continuous scheme	k_f, k_r, R_1, R_2, P	$k_\mathrm{f} \cdot R_1 \cdot R_2 - k_\mathrm{r} \cdot P^3$
0000107	Second order forward with two reactants, third order reverse with two products, reversible mass action kinetics, continuous scheme	k_f, k_r, R_1, R_2, P_1, P_2	$k_\mathrm{f} \cdot R_1 \cdot R_2 - k_\mathrm{r} \cdot P_1^2 \cdot P_2$
0000108	Second order forward with two reactants, third order reverse with three products, reversible mass action kinetics, continuous scheme	k_f, k_r, R_1, R_2, P_1, P_2, P_3	$k_\mathrm{f} \cdot R_1 \cdot R_2 - k_\mathrm{r} \cdot P_1 \cdot P_2 \cdot P_3$
**0000109**	Third order forward reversible mass action kinetics	general category	
**0000110**	Third order forward with two reactants reversible mass action kinetics	general category	
0000111	Third order forward with two reactants, zeroth order reverse, reversible mass action kinetics, continuous scheme	k_f, k_r, R_1, R_2	$k_\mathrm{f} \cdot R_1^2 \cdot R_2 - k_\mathrm{r}$
0000112	Third order forward with two reactants, first order reverse, reversible mass action kinetics, continuous scheme	k_f, k_r, R_1, R_2, P	$k_\mathrm{f} \cdot R_1^2 \cdot R_2 - k_\mathrm{r} \cdot P$
**0000113**	Third order forward with two reactants, second order reverse, reversible mass action kinetics	general category	
0000114	Third order forward with two reactants, second order reverse with one product, reversible mass action kinetics, continuous scheme	k_f, k_r, R_1, R_2, P	$k_\mathrm{f} \cdot R_1^2 \cdot R_2 - k_\mathrm{r} \cdot P^2$
0000115	Third order forward with two reactants, second order reverse with two products, reversible mass action kinetics, continuous scheme	k_f, k_r, R_1, R_2, P_1, P_2	$k_\mathrm{f} \cdot R_1^2 \cdot R_2 - k_\mathrm{r} \cdot P_1 \cdot P_2$

SBO	Term	Variables	Formula
**0000116**	Third order forward with two reactants, third order reverse, reversible mass action kinetics	general category	
0000117	Third order forward with two reactants, third order reverse with one product, reversible mass action kinetics, continuous scheme	k_f, k_r, R_1, R_2, P	$k_\mathrm{f} \cdot R_1^2 \cdot R_2 - k_\mathrm{r} \cdot P \cdot P^2$
0000118	Third order forward with two reactants, third order reverse with two products, reversible mass action kinetics, continuous scheme	k_f, k_r, R_1, R_2, P_1, P_2	$k_\mathrm{f} \cdot R_1^2 \cdot R_2 - k_\mathrm{r} \cdot P_1 \cdot P_1 \cdot P_2$
0000119	Third order forward with two reactants, third order reverse with three products, reversible mass action kinetics, continuous scheme	k_f, k_r, R_1, R_2, P_1, P_2, P_3	$k_\mathrm{f} \cdot R_1^2 \cdot R_2 - k_\mathrm{r} \cdot P_1 \cdot P_2 \cdot P_3$
**0000120**	Third order forward with three reactants reversible mass action kinetics	general category	
0000121	Third order forward with three reactants, zeroth order reverse, reversible mass action kinetics, continuous scheme	k_f, k_r, R_1, R_2, R_3	$k_\mathrm{f} \cdot R_1 \cdot R_2 \cdot R_3 - k_\mathrm{r}$
0000122	Third order forward with three reactants, first order reverse, reversible mass action kinetics, continuous scheme	k_f, k_r, R_1, R_2, R_3, P	$k_\mathrm{f} \cdot R_1 \cdot R_2 \cdot R_3 - k_\mathrm{r} \cdot P$
**0000123**	Third order forward with three reactants, second order reverse, reversible mass action kinetics	general category	
0000124	Third order forward with three reactants, second order reverse with one product, reversible mass action kinetics, continuous scheme	k_f, k_r, R_1, R_2, R_3, P	$k_\mathrm{f} \cdot R_1 \cdot R_2 \cdot R_3 - k_\mathrm{r} \cdot P^2$
0000125	Third order forward with three reactants, second order reverse with two products, reversible mass action kinetics, continuous scheme	k_f, k_r, R_1, R_2, R_3, P_1, P_2	$k_\mathrm{f} \cdot R_1 \cdot R_2 \cdot R_3 - k_\mathrm{r} \cdot P_1 \cdot P_2$

SBO	Term	Variables	Formula
**0000126**	Third order forward with three reactants, third order reverse, reversible mass action kinetics	general category	
0000127	Third order forward with three reactants, third order reverse with one product, reversible mass action kinetics, continuous scheme	k_f, k_r, R_1, R_2, R_3, P	$k_\mathrm{f} \cdot R_1 \cdot R_2 \cdot R_3 - k_\mathrm{r} \cdot P^3$
0000128	Third order forward with three reactants, third order reverse with two products, reversible mass action kinetics, continuous scheme	k_f, k_r, R_1, R_2, R_3, P_1, P_2	$k_\mathrm{f} \cdot R_1 \cdot R_2 \cdot R_3 - k_\mathrm{r} \cdot P_1^2 \cdot P_2$
0000129	Third order forward with three reactants, third order reverse with three products, reversible mass action kinetics, continuous scheme	k_f, k_r, R_1, R_2, R_3, P_1, P_2, P_3	$k_\mathrm{f} \cdot R_1 \cdot R_2 \cdot R_3 - k_\mathrm{r} \cdot P_1 \cdot P_2 \cdot P_3$
**0000130**	Third order forward with one reactant reversible mass action kinetics	general category	
0000131	Third order forward with one reactant, zeroth order reverse, reversible mass action kinetics, continuous scheme	$k_\mathrm{f}, k_\mathrm{r}, R$	$k_\mathrm{f} \cdot R^3 - k_\mathrm{r}$
0000132	Third order forward with one reactant, first order reverse, reversible mass action kinetics, continuous scheme	k_f, k_r, R, P	$k_\mathrm{f} \cdot R^3 - k_\mathrm{r} \cdot P$
**0000133**	Third order forward with one reactant, second order reverse, reversible mass action kinetics	general category	
0000134	Third order forward with one reactant, second order reverse with one product, reversible mass action kinetics, continuous scheme	$k_\mathrm{f}, k_\mathrm{r}, R, P$	$k_\mathrm{f} \cdot R^3 - k_\mathrm{r} \cdot P^2$
0000135	Third order forward with one reactant, second order reverse with two products, reversible mass action kinetics, continuous scheme	$k_\mathrm{f}, k_\mathrm{r}, R, P_1$, P_2	$k_\mathrm{f} \cdot R^3 - k_\mathrm{r} \cdot P_1 \cdot P_2$

SBO	Term	Variables	Formula
**0000136**	Third order forward with one reactant, third order reverse, reversible mass action kinetics	general category	
0000137	Third order forward with one reactant, third order reverse with one product, reversible mass action kinetics, continuous scheme	$k_\mathrm{f}, k_\mathrm{r}, R, P$	$k_\mathrm{f} \cdot R^3 - k_\mathrm{r} \cdot P^3$
0000138	Third order forward with one reactant, third order reverse with two products, reversible mass action kinetics, continuous scheme	$k_\mathrm{f}, k_\mathrm{r}, R, P_1, P_2$	$k_\mathrm{f} \cdot R^3 - k_\mathrm{r} \cdot P_1 \cdot P_2$
0000139	Third order forward with one reactant, third order reverse with three products, reversible mass action kinetics, continuous scheme	$k_\mathrm{f}, k_\mathrm{r}, R, P_1, P_2, P_3$	$k_\mathrm{f} \cdot R^3 - k_\mathrm{r} \cdot P_1 \cdot P_2 \cdot P_3$
**0000192**	Hill equation	V_max, R, K, h	$\frac{V_\mathrm{max} \cdot R^h}{K+R^h}$
0000195	Hill equation, microscopic form	V_max, R, K, h	$\frac{V_\mathrm{max} \cdot R^h}{K^h+R^h}$
0000198	Hill equation, reduced form	V_max, $R*$, K, h	$\frac{V_\mathrm{max} \cdot R*^h}{1+R*^h}$
**0000268**	Enzyme kinetics	general category	
**0000150**	Kinetics of irreversible non-modulated non-interacting reactant enzymes	E, k_+, n, S, K	$\frac{E \cdot k_+ \cdot \prod_{i=1}^n \frac{S_i}{K_i}}{\prod_{i=1}^n \left(1+\frac{S_i}{K_i}\right)}$
**0000028**	Kinetics of irreversible non-modulated unireactant enzymes	k_cat, E_T, S, K_S	$\frac{k_\mathrm{cat} \cdot E_\mathrm{T} \cdot S}{K_\mathrm{S}+S}$
0000029	Henri-Michaelis-Menten equation	k_cat, E_T, S, K_S	$\frac{k_\mathrm{cat} \cdot E_\mathrm{T} \cdot S}{K_\mathrm{S}+S}$
0000030	Van Slyke-Cullen equation	k_cat, E_T, S, K_S	$\frac{k_\mathrm{cat} \cdot E_\mathrm{T} \cdot S}{K_\mathrm{S}+S}$
0000031	Briggs-Haldane equation	k_cat, E_T, S, K_M	$\frac{k_\mathrm{cat} \cdot E_\mathrm{T} \cdot S}{K_\mathrm{M}+S}$
0000199	Normalized kinetics of unireactant enzymes	$k_\mathrm{cat}, S, K_\mathrm{S}$	$\frac{k_\mathrm{cat} \cdot S}{K_\mathrm{S}+S}$
0000151	Kinetics of irreversible non-modulated non-interacting bireactant enzymes	E, k_+, n, S_1, S_2, K_1, K_2	$\frac{E \cdot k_+ \cdot \frac{S_1}{K_1} \cdot \frac{S_2}{K_2}}{\left(1+\frac{S_1}{K_1}\right) \cdot \left(1+\frac{S_2}{K_2}\right)}$

SBO	Term	Variables	Formula
0000152	Kinetics of irreversible non-modulated non-interacting trireactant enzymes	E, k_+, n, S_1, S_2, S_3, K_1, K_2, K_3	$\frac{E\cdot k_+\cdot\frac{S_1}{K_1}\cdot\frac{S_2}{K_2}\cdot\frac{S_3}{K_3}}{\left(1+\frac{S_1}{K_1}\right)\cdot\left(1+\frac{S_2}{K_2}\right)\cdot\left(1+\frac{S_3}{K_3}\right)}$
**0000269**	Kinetics of unireactant enzymes	general category	
0000262	Simple uncompetitive inhibition of irreversible unireactant enzymes	k_{cat}, E_{T}, S, I, K_{S}, K_{I}	$\frac{k_{\mathrm{cat}}\cdot E_{\mathrm{T}}\cdot S}{S\cdot\left(1+\frac{I}{K_{\mathrm{I}}}\right)+K_{\mathrm{S}}}$
**0000270**	Competitive inhibition of irreversible unireactant enzymes by exclusive inhibitors	k_{cat}, E_{T}, S, I, K_{S}, K_{I}, n	$\frac{k_{\mathrm{cat}}\cdot E_{\mathrm{T}}\cdot S}{K_{\mathrm{S}}\cdot\left(1+\sum_{i=1}^{n}\frac{I_i}{K_{\mathrm{I}i}}\right)+S}$
0000260	Simple competitive inhibition of irreversible unireactant enzymes by one inhibitor	k_{cat}, E_{T}, S, I, K_{S}, K_{I}	$\frac{k_{\mathrm{cat}}\cdot E_{\mathrm{T}}\cdot S}{K_{\mathrm{S}}\cdot\left(1+\frac{I}{K_{\mathrm{I}}}\right)+S}$
0000271	Competitive inhibition of irreversible unireactant enzymes by two exclusive inhibitors	k_{cat}, E_{T}, S, I_1, I_2, K_{S}, K_{I1}, K_{I2}, K_{I2}	$\frac{k_{\mathrm{cat}}\cdot E_{\mathrm{T}}\cdot S}{K_{\mathrm{S}}\cdot\left(1+\frac{I_1}{K_{\mathrm{I1}}}+\frac{I_2}{K_{\mathrm{I2}}}\right)+S}$
**0000273**	Competitive inhibition of irreversible unireactant enzymes by non-exclusive non-cooperative inhibitors	k_{cat}, E_{T}, S, I, K_{S}, K_{I}, n, m	$\frac{k_{\mathrm{cat}}\cdot E_{\mathrm{T}}\cdot S}{K_{\mathrm{S}}\cdot\prod_{i=1}^{n}\left(1+\frac{I_i}{K_{\mathrm{I}i}}\right)^{m_i}+S}$
**0000267**	Competitive inhibition of irreversible unireactant enzymes by one inhibitor	k_{cat}, E_{T}, S, I, K_{S}, K_{I}, n	$\frac{k_{\mathrm{cat}}\cdot E_{\mathrm{T}}\cdot S}{S+K_{\mathrm{S}}\cdot\left(1+\frac{I}{K_{\mathrm{I}}}\right)^{n}}$
**0000274**	Simple competitive inhibition of irreversible unireactant enzymes by two non-exclusive inhibitors	k_{cat}, E_{T}, S, I_1, I_2, K_{S}, K_{I1}, K_{I2}	$\frac{k_{\mathrm{cat}}\cdot E_{\mathrm{T}}\cdot S}{K_{\mathrm{S}}\cdot\left(1+\frac{I_1}{K_{\mathrm{I1}}}+\frac{I_2}{K_{\mathrm{I2}}}\right)+S}$
**0000275**	Mixed-type inhibition of irreversible enzymes by mutually exclusive inhibitors	k_{cat}, E_{T}, S, I, K_{S}, K_{I}, a, n	$\frac{k_{\mathrm{cat}}\cdot E_{\mathrm{T}}\cdot S}{K_{\mathrm{S}}\cdot\left(1+\sum_{i=1}^{n}\frac{I_i}{K_{\mathrm{I}i}}\right)+S\cdot\left(1+\sum_{i=0}^{n}\frac{I_i}{a_i\cdot K_{\mathrm{I}i}}\right)}$
**0000265**	Simple mixed-type inhibition of irreversible unireactant enzymes	k_{cat}, E_{T}, S, I, K_{S}, K_{I}, a	$\frac{k_{\mathrm{cat}}\cdot E_{\mathrm{T}}\cdot S}{S\cdot\left(1+\frac{I}{a\cdot K_{\mathrm{I}}}\right)+K_{\mathrm{S}}\cdot\left(1+\frac{I}{K_{\mathrm{I}}}\right)}$
0000266	Simple non-competitive inhibition of unireactant enzymes	k_{cat}, E_{T}, S, I, K_{S}, K_{I}	$\frac{k_{\mathrm{cat}}\cdot E_{\mathrm{T}}\cdot S}{S\cdot\left(1+\frac{I}{K_{\mathrm{I}}}\right)+K_{\mathrm{S}}\cdot\left(1+\frac{I}{K_{\mathrm{I}}}\right)}$
**0000276**	Mixed-type inhibition of irreversible unireactant enzymes by two inhibitors	k_{cat}, E_{T}, S, I_1, I_2, K_{S}, K_{I1}, K_{I2}, a, b	$\frac{k_{\mathrm{cat}}\cdot E_{\mathrm{T}}\cdot S}{S\cdot\left(1+\frac{I_1}{a\cdot K_{\mathrm{I1}}}+\frac{I_2}{b\cdot K_{\mathrm{I2}}}\right)+K_{\mathrm{S}}\cdot\left(1+\frac{I}{K_{\mathrm{I1}}}+\frac{I_2}{K_{\mathrm{I2}}}\right)}$

SBO	Term	Variables	Formula
0000277	Mixed-type inhibition of unireactant enzymes by two inhibitors	k_{cat}, E_{T}, S, I_1, I_2, K_{S}, K_{I1}, K_{I2}	$\frac{k_{\mathrm{cat}}\cdot E_{\mathrm{T}}\cdot S}{S\cdot\left(1+\frac{I_1}{K_{\mathrm{I1}}}+\frac{I_2}{K_{\mathrm{I2}}}\right)+K_{\mathrm{S}}\cdot\left(1+\frac{I_1}{K_{\mathrm{I1}}}+\frac{I_2}{K_{\mathrm{I2}}}\right)}$
**0000326**	Kinetics of non-modulated unireactant enzymes	$k_{-\mathrm{cat}}$, $k_{+\mathrm{cat}}$, E_{T}, S, P, K_{MS}, K_{MP}	$\frac{k_{+\mathrm{cat}}\cdot E_{\mathrm{T}}\cdot\frac{S}{K_{\mathrm{MS}}}-k_{-\mathrm{cat}}\cdot E_{\mathrm{T}}\cdot\frac{P}{K_{\mathrm{MP}}}}{1+\frac{S}{K_{\mathrm{MS}}}+\frac{P}{K_{\mathrm{MP}}}}$

References

[1] Hucka M, Finney A, Sauro HM, Bolouri H, Doyle JC, Kitano H, Arkin AP, Bornstein BJ, Bray D, Cornish-Bowden A, Cuellar AA, Dronov S, Gilles ED, Ginkel M, Gor V, Goryanin II, Hedley WJ, Hodgman TC, Hofmeyr JH, Hunter PJ, Juty NS, Kasberger JL, Kremling A, Kummer U, Le Novere N, Loew LM, Lucio D, Mendes P, Minch E, Mjolsness ED, Nakayama Y, Nelson MR, Nielsen PF, Sakurada T, Schaff JC, Shapiro BE, Shimizu TS, Spence HD, Stelling J, Takahashi K, Tomita M, Wagner J, Wang J: **The systems biology markup language (SBML): a medium for representation and exchange of biochemical network models**. *Bioinformatics* 2003, **19**(4):524–531, [`http://bioinformatics.oxfordjournals.org/cgi/content/abstract/19/4/524`].

[2] Cornish-Bowden A: *Fundamentals of Enzyme Kinetics*. 59 Portland Place, London: Portland Press Ltd., 3rd edition 2004.

[3] Segel IH: *Enzyme Kinetics—Behavior and Analysis of Rapid Equilibrium and Steady-State Enzyme Systems*. Wiley Classics Library Edition 1993.

[4] Liebermeister W, Klipp E: **Bringing metabolic networks to life: convenience rate law and thermodynamic constraints**. *Theor Biol Med Model* 2006, **3**:41, [`http://dx.doi.org/10.1186/1742-4682-3-41`].

[5] Kitano H, Funahashi A, Matsuoka Y, Oda K: **Using process diagrams for the graphical representation of biological networks**. *Nature Biotechnology* 2005, **23**(8):961–966, [`http://dx.doi.org/10.1038/nbt1111`].

[6] **CellDesigner.org** [`http://celldesigner.org`].

[7] Funahashi A, Tanimura N, Morohashi M, Kitano H: **CellDesigner: a process diagram editor for gene-regulatory and biochemical networks**. *BioSilico* 2003, **1**(5):159–162, [`http://www.symbio.jst.go.jp/symbio/paper/BioSilicoNov2003Funahashi.pdf`].

[8] **SBMLsqueezer Project Home Page** [`http://www.ra.cs.uni-tuebingen.de/software/SBMLsqueezer`].

[9] **SBML—Systems Biology Markup Language** [`http://sbml.org`].

[10] Dräger A, Supper J, Planatscher H, Magnus JB, Oldiges M, Zell A: **Comparing Various Evolutionary Algorithms on the Parameter Optimization of the Valine and Leucine Biosynthesis in *Corynebacterium glutamicum***. In *2007 IEEE Congress on Evolutionary Computation*.

Edited by Srinivasan D, Wang L, IEEE Computational Intelligence Society, Singapore: IEEE Press 2007:620–627.

[11] Heinrich R, Schuster S: *The Regulation of Cellular Systems*. 115 Fifth Avenue New York, NY 10003: Chapman and Hall 1996.

[12] Dräger A, Kronfeld M, Supper J, Planatscher H, Magnus JB, Oldiges M, Zell A: **Benchmarking Evolutionary Algorithms on Convenience Kinetics Models of the Valine and Leucine Biosynthesis in *C. glutamicum***. In *2007 IEEE Congress on Evolutionary Computation*. Edited by Srinivasan D, Wang L, IEEE Computational Intelligence Society, Singapore: IEEE Press 2007:896–903.

[13] Hinze T, Hayat S, Lenser T, Matsumaru N, Dittrich P: **Hill Kinetics meets P Systems: A Case Study on Gene Regulatory Networks as Computing Agents *in silico* and *in vivo***. In *Proceedings of the Eighth Workshop on Membrane Computing*. Edited by Eleftherakis G, Kefalas P, Paun G, SEERC 2007:363–381.

[14] Bisswanger H: *Enzymkinetik – Theorie und Methoden*. Weinheim, Germany: Wiley-VCH, 3rd edition 2000.

[15] **SBO::Systems Biology Ontology** [`http://www.ebi.ac.uk/sbo/`].
