# Supplementary material for: SBMLsqueezer: A CellDesigner plug-in to generate kinetic rate equations for biochemical networks
Source: BMC Syst Biol. 2008 Apr 30;2:39. doi: 10.1186/1752-0509-2-39 (PMC2412839; doi:10.1186/1752-0509-2-39)
Supplement: Additional file 2 — SBMLsqueezer: Tutorial. This tutorial provides installation instructions and guides the user through a real-world example of a T-cell signaling network. It highlights the modeling capabilities and the application of SBMLsqueezer to create a quantitative biochemical model. Finally it is shown how to apply CellDesigner's simulation module to the network to produce a dynamic simulation of the temporal changes of the species' concentrations. [file 1752-0509-2-39-S2.pdf]

EBERHARD-KARLS-UNIVERSITÄT TÜBINGEN
Wilhelm-Schickard-Institut für Informatik
Lehrstuhl Rechnerarchitektur

Supplementary Material

SBMLsqueezer: Tutorial

Andreas Dräger* Nadine Hassis Jochen Supper
Adrian Schröder Andreas Zell

April 28, 2008

*Corresponding author, e-mail: `andreas.draeger@uni-tuebingen.de`

SBMLsqueezer is a CellDesigner plug-in for the fast generation of kinetic equations to model the dynamic behavior of complex biochemical reaction networks. Based on the Systems Biology Graphical Notation (SBGN) scheme used by CellDesigner, SBMLsqueezer is able to recognize special reaction types and to generate appropriate mathematical equations to model each reaction automatically. The user can choose between two modes to use SBMLsqueezer for modeling: (i) SBMLsqueezer can read in a network topology and generate the whole system of differential equations at once with respect to user settings. (ii) All equations can be generated one by one using CellDesigner's reaction context menu. When applying the second approach, SBMLsqueezer analyzes the desired reaction for its properties and offers a predefined list of applicable rate laws. All mathematical equations and associated kinetic parameters are accessible through the designated menu items in the CellDesigner user interface. Furthermore, an export function allows writing all equations of the system into a LaTeX or plain text file for scientific writing and further processing.

Contents

1 Generation of Kinetic Laws for a T-cell Signaling Network in Three Steps 4
1.1 Installation of the SBMLsqueezer Plug-in for CellDesigner 4
1.2 Open the Example Topology “TCellSignaltransduction.xml” 5
1.3 Use SBMLsqueezer to Generate Kinetic Laws 5
1.3.1 Generate each Equation One by One 5
1.3.2 Generate an Equation System for a Whole Topology in One Go 5

2 Modeling Single Reactions of the T-cell Signaling Network One by One 8
2.1 Irreversible/Reversible Association Reaction 8
2.2 Internalization 8
2.3 Single Enzyme-Catalyzed Reaction 9
2.4 Dual Enzyme-Catalyzed Reactions 9

1 Generation of Kinetic Laws for a T-cell Signaling Network in Three Steps

SBMLsqueezer offers two different approaches for automatically generating kinetic equations for biochemical reactions within CellDesigner. First, each reaction of a given network can be modeled individually. Second, the whole equation system for a given reaction network can be generated in one go. In the first case, the user needs to right-click on the each reaction and to choose SBMLsqueezer in the emerging context menu (Figure 1(a)). In the latter case, the user needs to call up SBMLsqueezer through the “Plugin”-menu in the task bar (Figure 1(b)).

To explain both modeling strategies, this tutorial takes a model of the T-cell signal transduction pathway as a real-life example. This signal cascade is initialized with the stimulation of CD3/CD28 and ends with the translocation of the nuclear factor of activated T-cells (NFAT). The SBML model of this network can be downloaded from the project web page.

The remainder of this section gives a rough introduction to SBMLsqueezer and illustrates the generation of kinetic equations in three main steps. In Section 2 the kinetic modeling of individual reactions with different types of rate laws is discussed in more detail. The kinetic laws provided by SBMLsqueezer are explained in detail in the Kinetic Laws supplement.

1.1 Installation of the SBMLsqueezer Plug-in for CellDesigner

- Requirements:
  - CellDesigner 4.0 or higher
  - Java 1.5
- Installation of CellDesigner 4.0.
- Download SBMLsqueezer from the project web page.
- Copy the SBMLsqueezer jar-file into the plug-in directory of CellDesigner. For instance
  `/opt/CellDesigner4.0/plugin/` under Linux or
  `C:\Programs\CellDesigner\plugin\` under Windows

1.2 Open the Example Topology “TCellSignaltransduction.xml”

- Download the example SBML file from the project web page.
- Open “TCellSignaltransduction.xml” with CellDesigner.

1.3 Use SBMLsqueezer to Generate Kinetic Laws

SBMLsqueezer offers two ways to create rate laws for a given model. Both are described in the following. Once kinetic equations have been assigned to each reaction in the system, a dynamic simulation can be performed. To use this feature in CellDesigner, one has to select “Simulate” → “Control Panel” → “Execute” and a dialog window appears, which provides several settings for the simulation (for details, see the documentation of CellDesigner and Figure 5).

1.3.1 Generate each Equation One by One

Right-click on the desired reaction arrow and choose “SBMLsqueezer” → “Squeeze Kinetic Law” (Figure 1(a)).

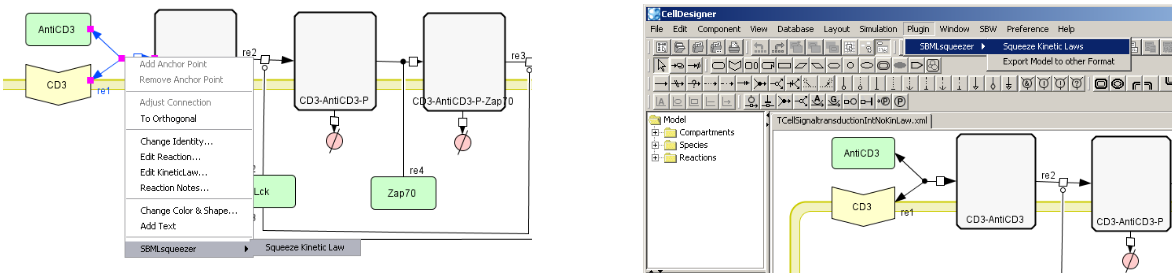

(a) Open SBMLsqueezer through the context menu (b) Open SBMLsqueezer through the task bar

Figure 1: Open SBMLsqueezer

1.3.2 Generate an Equation System for a Whole Topology in One Go

- Click on “Plugin” → “SBMLsqueezer” → “Squeeze Kinetic Laws” (Figures 1(b) and 2). Alternatively, the model can also be exported to LaTeX by clicking on “Export Model to other Format”.
- Click the “Generate” button to generate kinetic equations using the SBMLsqueezer default options (Figure 4). Generated equations can be browsed within the SBMLsqueezer Graphical User Interface (GUI).
- The button “Help” opens a help browser providing additional information and a link to the project website.
- Click the “Apply” button to transfer the generated equations to the SBML-file.
- After the “Apply” button has been clicked, all kinetic equations can be found within CellDesigner’s “Reactions” table.

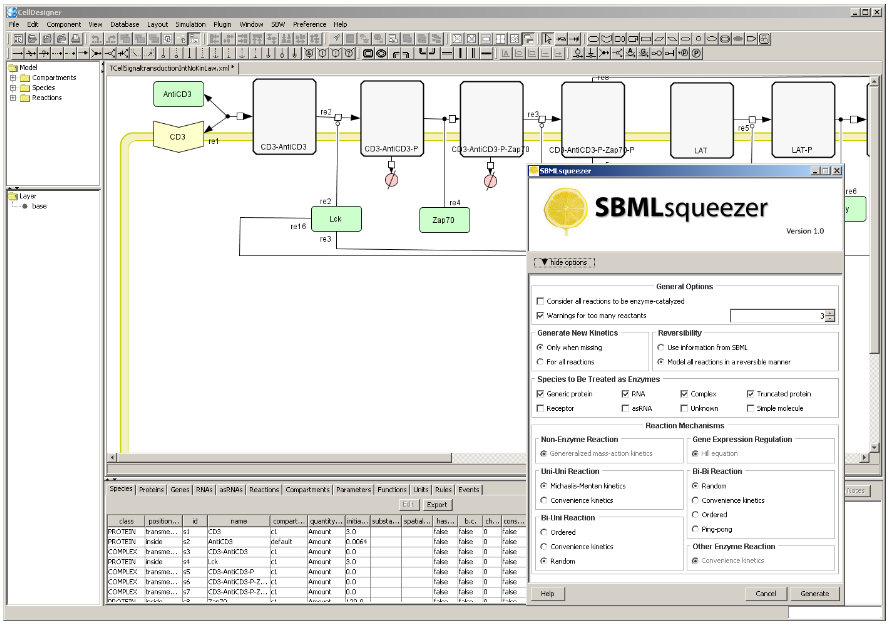

Figure 2: SBMLsqueezer dialog

After starting SBMLsqueezer one can browse the “Options Menu” by clicking on “show options”. This menu provides the opportunity to perform several changes to the default settings. The meaning of all options is explained in detail in the paragraph Adjusting the Preferences.

- Alternatively, all equations within the SBML model can also be saved in LaTeX or plain ASCII text format by clicking on the “Save” button.
- All parameter values are stored within the “Parameters” table.
- Parameter values are initiated with 1.0 per default.

Adjusting the Preferences

General Options In this section the user can specify if catalysts must be added to each reaction explicitly or if for the sake of simplicity all reactions are considered to be enzyme-catalyzed. The second checkbox asks whether warnings should occur if the number of participating reactants exceeds a given threshold. By default, more than three reactants is considered unrealistic.

Generate New Kinetics The user can specify whether equations should be generated for all reactions or only missing ones should be assigned to the respective reactions. By default, SBMLsqueezer creates new kinetic equations only for reactions which currently lack such a law. The user can also force it to overwrite all existing kinetics by selecting “For all reactions”.

Reversibility If desired, all reactions can be set to reversible and the rate laws are created accordingly. Therefore, the user can select the default “Model all reactions in a reversible manner”. Otherwise, SBMLsqueezer can preserve the reactions reversible/irreversible flag, as given in the model.

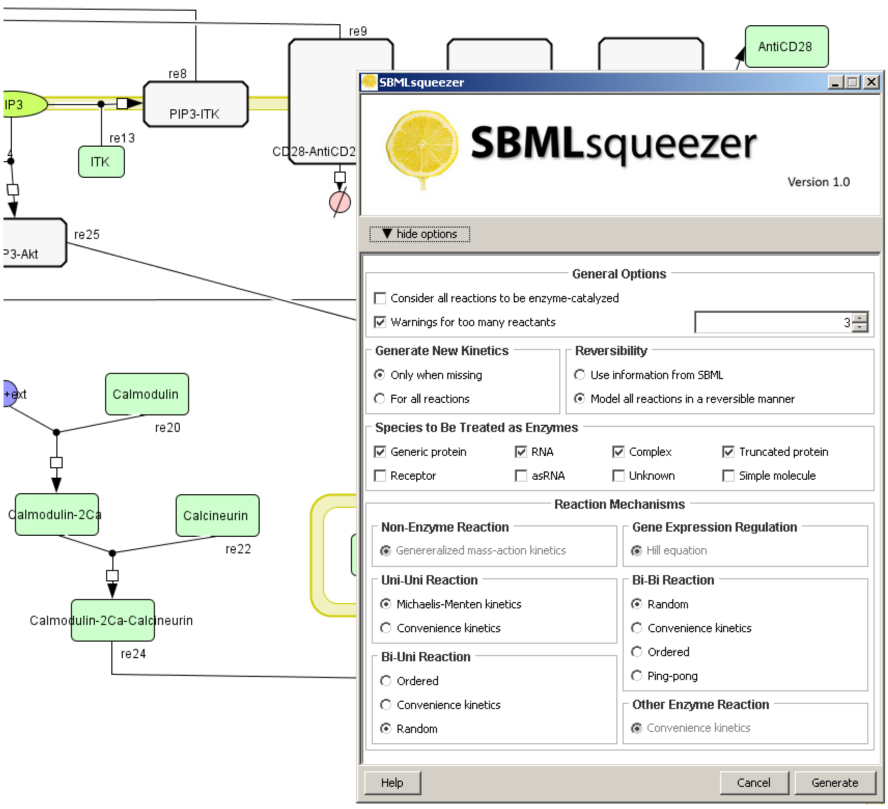

Figure 3: Options dialog in detail

Species to Be Treated as Enzymes Since it is not always clear in the current version of CellDesigner which catalyst can act as an enzyme, this section allows selecting those species that may be considered as enzymes when acting as catalysts. By default these are “Generic protein”, “RNA”, “Complex” and “Truncated protein”. The user may also select “Receptor”, “asRNA”, “Unknown” or “Simple molecule” or deselect any one of the preselected types. A reaction catalyzed by a species which is not an enzyme will be modeled using generalized mass-action kinetics.

Reaction Mechanisms This section allows the user to specify the type of kinetic equation to be applied for each specific type of reaction. There is currently no alternative kinetic for “Non-Enzyme Reaction”, since those reactions are always modeled using generalized mass-action kinetics, and “Gene Expression Regulation”, for which the Hill equation is always applied, as well as for any “Other Enzyme Reaction” which doesn’t fall into the categories “Uni-Uni”, “Bi-Bi” and “Bi-Uni”. Only the semi-mechanistic convenience rate law is capable of describing these more complex reactions without detailed knowledge of the underlying reaction mechanism. All other types are basic reaction mechanisms, and the user can choose the mechanism of each reaction. Note that changes to the generated equations can be made subsequently by double-clicking on the name of the rate law in the result table (Figure 4).

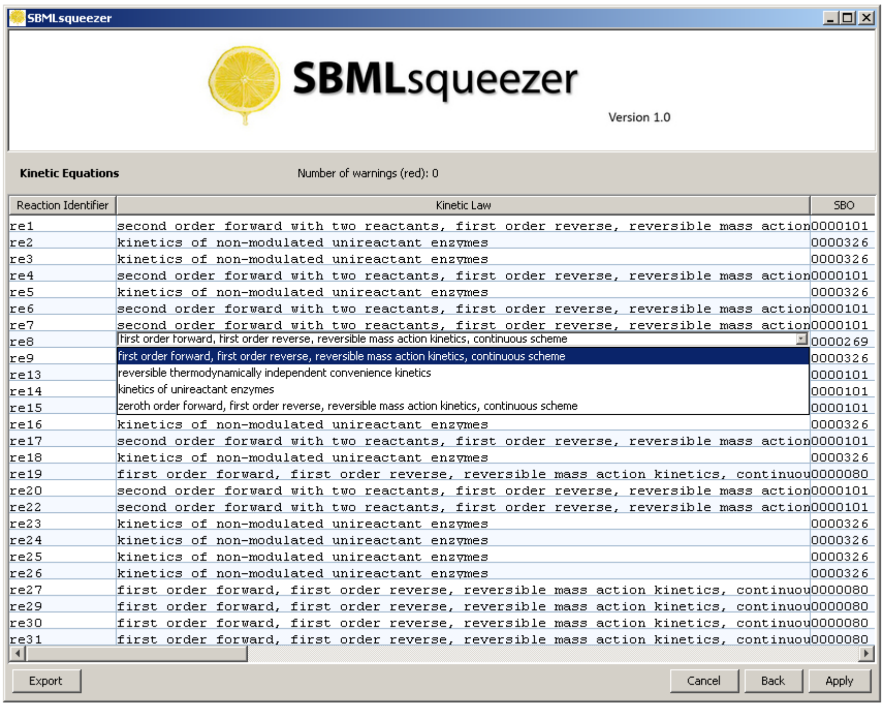

Figure 4: Apply reactions

After pushing the “Generate” button all generated equations are listed, clearly arranged in an editable table. If desired, further modifications to the equations can be made through this interface. By clicking the “Apply” button, all equations and corresponding kinetic parameters are stored in the designated tables within CellDesigner. All kinetic parameters are initialized with 1.0 and can be modified manually. By pushing “File” → “Save” all modifications are stored in the SBML-file.

2 Modeling Single Reactions of the T-cell Signaling Network One by One

Rate equations can also be assigned one by one to each reaction within a model. For a better visualization, SBMLsqueezer uses the HOTEQN library to provide a preview of each generated equation.

2.1 Irreversible/Reversible Association Reaction

The first example shows the simple binding of a stimulatory antibody at the CD3 receptor of a T-cell. SBMLsqueezer suggests six different rate laws for modeling this simple association reaction within the context menu. Each rate law can be modeled in a reversible or irreversible manner. The results for each case when using the convenience kinetics are shown in Figure 6.

2.2 Internalization

T-cell receptors are subjected to internalization if they have been bound to pMHC and triggering events were activated. This process can be modeled using generalized mass-action kinetics. The user can choose between the irreversible and the reversible form of this rate law (see Figure 7). Alternatively, one may also apply a zeroth order rate law.

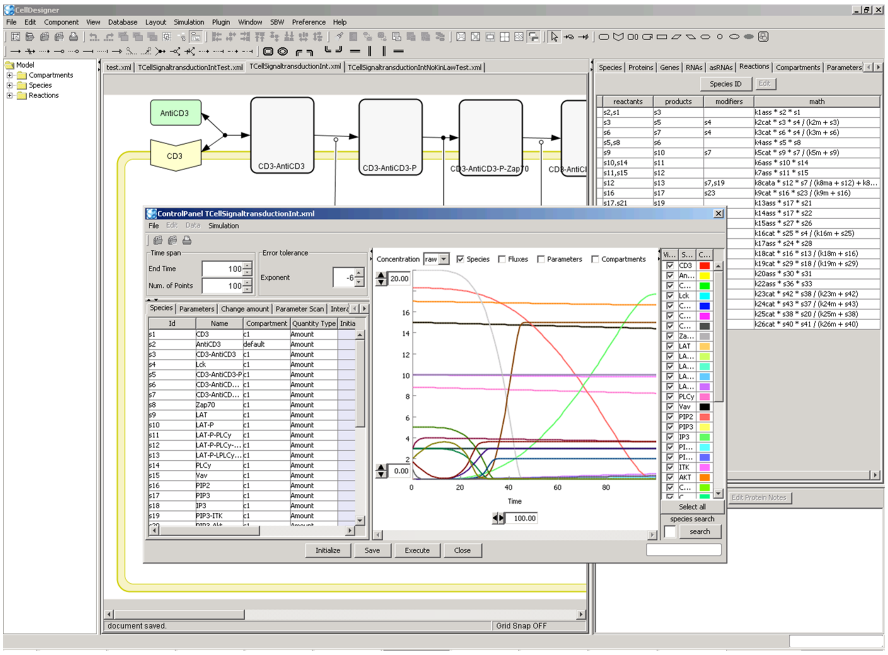

Figure 5: Dynamic simulation

After all equations are generated with SBMLsqueezer the whole network can be simulated dynamically by pushing “Simulation” → “Execute”.

2.3 Single Enzyme-Catalyzed Reaction

SBMLsqueezer suggests four different rate laws to model simple enzyme reactions (Figure 8). First, generalized mass-action kinetics, second, convenience kinetics and third Michaelis-Menten kinetics. As a fourth alternative, zeroth order mass-action kinetics may be applied as well. Each of the three kinetics can again be modeled reversibly or irreversibly.

2.4 Dual Enzyme-Catalyzed Reactions

More complex enzyme-catalyzed reactions can also be modeled with the help of SBMLsqueezer. The example shown here is a reaction catalyzed by two different enzymes. The results for the irreversible and reversible cases can be found in Figure 9.

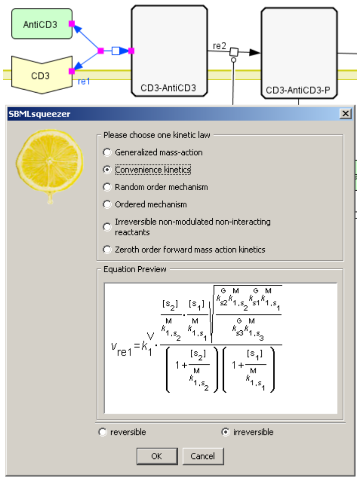

(a) Irreversible case

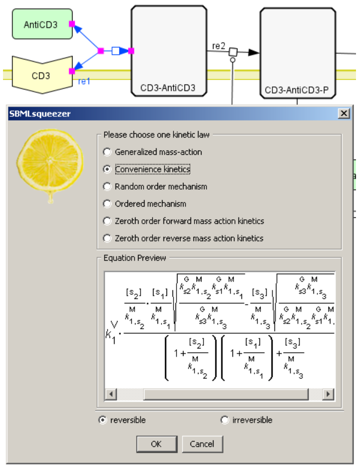

(b) Reversible case

$$v_{\text{irreversible}} = k_1^{\mathrm{V}} \cdot \frac{\frac{[s_2]}{k^{\mathrm{M}}_{1,s_2}} \cdot \frac{[s_1]}{k^{\mathrm{M}}_{1,s_1}} \sqrt{\frac{k^{\mathrm{G}}_{s2} k^{\mathrm{M}}_{1,s_2} k^{\mathrm{G}}_{s1} k^{\mathrm{M}}_{1,s_1}}{k^{\mathrm{G}}_{s3} k^{\mathrm{M}}_{1,s_3}}}}{\left(1 + \frac{[s_2]}{k^{\mathrm{M}}_{1,s_2}}\right)\left(1 + \frac{[s_1]}{k^{\mathrm{M}}_{1,s_1}}\right)} \tag{1}$$

$$v_{\text{reversible}} = k_1^{\mathrm{V}} \cdot \frac{\frac{[s_2]}{k^{\mathrm{M}}_{1,s_2}} \cdot \frac{[s_1]}{k^{\mathrm{M}}_{1,s_1}} \sqrt{\frac{k^{\mathrm{G}}_{s2} k^{\mathrm{M}}_{1,s_2} k^{\mathrm{G}}_{s1} k^{\mathrm{M}}_{1,s_1}}{k^{\mathrm{G}}_{s3} k^{\mathrm{M}}_{1,s_3}}} - \frac{[s_3]}{k^{\mathrm{M}}_{1,s_3}} \sqrt{\frac{k^{\mathrm{G}}_{s3} k^{\mathrm{M}}_{1,s_3}}{k^{\mathrm{G}}_{s2} k^{\mathrm{M}}_{1,s_2} k^{\mathrm{G}}_{s1} k^{\mathrm{M}}_{1,s_1}}}}{\left(1 + \frac{[s_2]}{k^{\mathrm{M}}_{1,s_2}}\right)\left(1 + \frac{[s_1]}{k^{\mathrm{M}}_{1,s_1}}\right) + \frac{[s_3]}{k^{\mathrm{M}}_{1,s_3}}} \tag{2}$$

(c) Irreversible and reversible formulas of an association reaction modeled with convenience kinetics. SBMLsqueezer uses the internal id of the species, i. e., $s_2 \equiv$ AntiCD3, $s_1 \equiv$ CD3, $s_3 \equiv$ CD3-AntiCD3

Figure 6: Association reaction

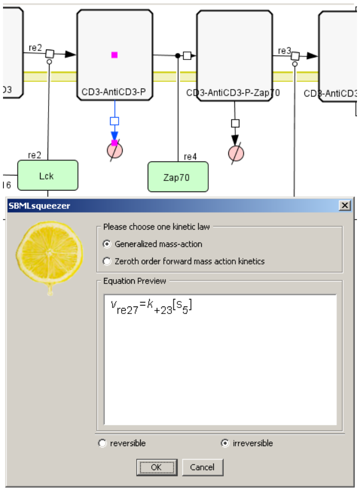

(a) Irreversible case

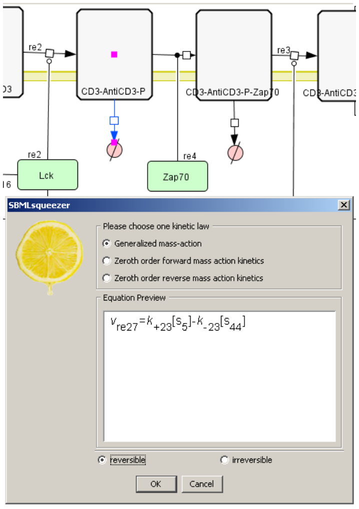

(b) Reversible case

$$v_{\text{irreversible}} = k_{+23}[\text{s}_5] \tag{3}$$

$$v_{\text{reversible}} = k_{+23}[\text{s}_5] - k_{-23}[\text{s}_{44}] \tag{4}$$

(c) Irreversible and reversible formulas of receptor internalization modeled with generalized mass-action kinetics. SBMLsqueezer uses the internal id of the species, i. e., $s_5 \equiv$ CD3-AntiCD3-P, $s_{44} \equiv$ Degradation

Figure 7: Internalization reaction

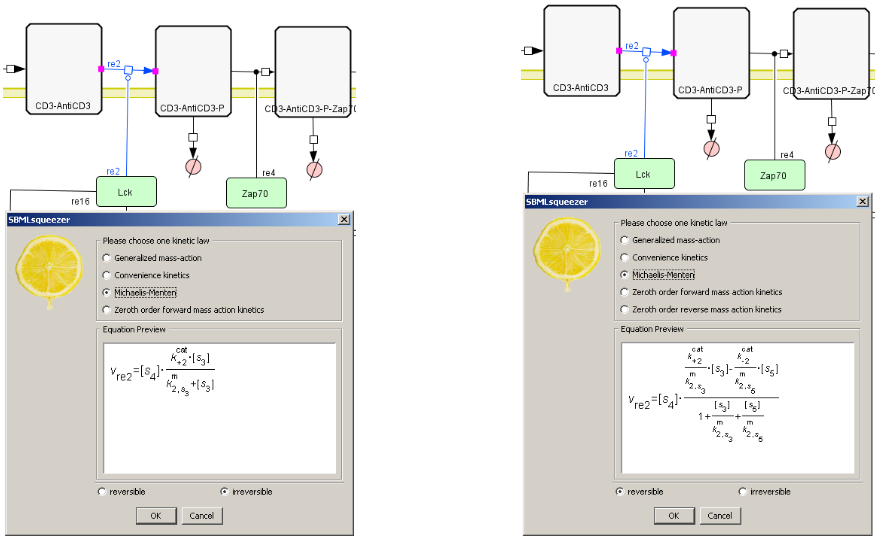

(a) Irreversible case

(b) Reversible case

$$v_{\text{irreversible}} = [\mathrm{s}_4] \cdot \frac{k^{\text{cat}}_{+2}[\mathrm{s}_3]}{k^{\mathrm{M}}_{2,\mathrm{s}_3} + [\mathrm{s}_3]} \tag{5}$$

$$v_{\text{reversible}} = [\mathrm{s}_4] \cdot \frac{\frac{k^{\text{cat}}_{+2}}{k^{\mathrm{M}}_{2,\mathrm{s}_3}}[\mathrm{s}_3] - \frac{k^{\text{cat}}_{-2}}{k^{\mathrm{M}}_{2,\mathrm{s}_5}}[\mathrm{s}_5]}{1 + \frac{[\mathrm{s}_3]}{k^{\mathrm{M}}_{2,\mathrm{s}_3}} + \frac{[\mathrm{s}_5]}{k^{\mathrm{M}}_{2,\mathrm{s}_5}}} \tag{6}$$

(c) Irreversible and reversible formulas of a single enzyme-catalyzed reaction modeled with Michaelis-Menten kinetics. SBMLsqueezer uses the internal id of the species, i. e., $s_4 \equiv$ Lck, $s_3 \equiv$ CD3-AntiCD3, $s_5 \equiv$ CD3-AntiCD3-P

Figure 8: Single enzyme-catalyzed reaction

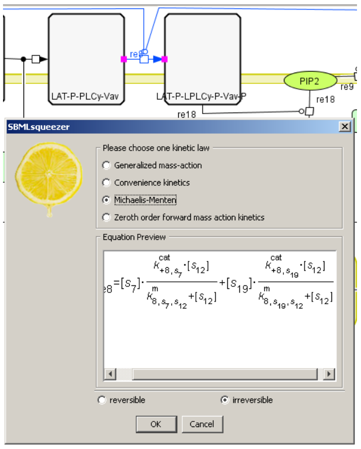

(a) Irreversible case

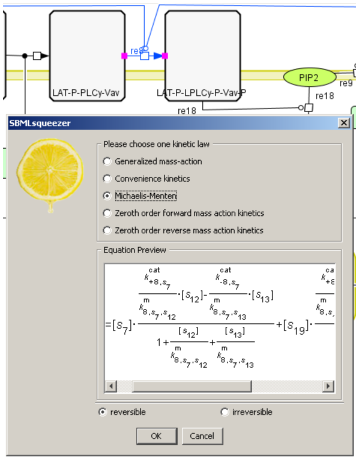

(b) Reversible case

$$v_{\text{irreversible}} = [\mathrm{s}_7] \cdot \frac{k^{\text{cat}}_{+8,\mathrm{s}_7}[\mathrm{s}_{12}]}{k^{\text{M}}_{8,\mathrm{s}_7,\mathrm{s}_{12}} + [\mathrm{s}_{12}]} + [\mathrm{s}_{19}] \cdot \frac{k^{\text{cat}}_{+8,\mathrm{s}_{19}}[\mathrm{s}_{12}]}{k^{\text{M}}_{8,\mathrm{s}_{19},\mathrm{s}_{12}} + [\mathrm{s}_{12}]} \tag{7}$$

$$v_{\text{reversible}} = [\mathrm{s}_7] \cdot \frac{\frac{k^{\text{cat}}_{+8,\mathrm{s}_7}}{k^{\text{M}}_{8,\mathrm{s}_7,\mathrm{s}_{12}}}[\mathrm{s}_{12}] - \frac{k^{\text{cat}}_{-8,\mathrm{s}_7}}{k^{\text{M}}_{8,\mathrm{s}_7,\mathrm{s}_{13}}}[\mathrm{s}_{13}]}{1 + \frac{[\mathrm{s}_{12}]}{k^{\text{M}}_{8,\mathrm{s}_7,\mathrm{s}_{12}}} + \frac{[\mathrm{s}_{13}]}{k^{\text{M}}_{8,\mathrm{s}_7,\mathrm{s}_{13}}}} + [\mathrm{s}_{19}] \cdot \frac{\frac{k^{\text{cat}}_{+8,\mathrm{s}_{19}}}{k^{\text{M}}_{8,\mathrm{s}_{19},\mathrm{s}_{12}}}[\mathrm{s}_{12}] - \frac{k^{\text{cat}}_{-8,\mathrm{s}_{19}}}{k^{\text{M}}_{8,\mathrm{s}_{19},\mathrm{s}_{13}}}[\mathrm{s}_{13}]}{1 + \frac{[\mathrm{s}_{12}]}{k^{\text{M}}_{8,\mathrm{s}_{19},\mathrm{s}_{12}}} + \frac{[\mathrm{s}_{13}]}{k^{\text{M}}_{8,\mathrm{s}_{19},\mathrm{s}_{13}}}} \tag{8}$$

(c) Irreversible and reversible formulas of a dual enzyme-catalyzed reaction modeled with Michaelis-Menten kinetics. SBMLsqueezer uses the internal id of the species, i. e., $s_{12} \equiv$ LAT-P-PLCγ-Vav, $s_{13} \equiv$ LAT-P-PLCγ-Vav-P, $s_{19} \equiv$ PIP3-ITK, $s_7 \equiv$ CD3-AntiCD3-P-Zap70

Figure 9: Dual enzyme-catalyzed reaction
